# Recognition of Membrane-Bound Fusion-Peptide/MPER Complexes by the HIV-1 Neutralizing 2F5 Antibody: Implications for Anti-2F5 Immunogenicity

**DOI:** 10.1371/journal.pone.0052740

**Published:** 2012-12-21

**Authors:** Nerea Huarte, Aitziber Araujo, Rocio Arranz, Maier Lorizate, Heribert Quendler, Renate Kunert, José M. Valpuesta, José L. Nieva

**Affiliations:** 1 Biophysics Unit (CSIC-UPV/EHU) and Biochemistry and Molecular Biology Department, University of the Basque Country (UPV/EHU), Bilbao, Spain; 2 Department of Macromolecular Structures, National Center for Biotechnology (CNB-CSIC), Madrid, Spain; 3 Institute of Applied Microbiology, University of Natural Resources and Life Sciences, Vienna, Austria; German Primate Center, Germany

## Abstract

The membrane proximal external region (MPER) of the fusogenic HIV-1 glycoprotein-41 harbors the epitope sequence recognized by 2F5, a broadly neutralizing antibody isolated from an infected individual. Structural mimicry of the conserved MPER 2F5 epitope constitutes a pursued goal in the field of anti-HIV vaccine development. It has been proposed that 2F5 epitope folding into its native state is attained in the vicinity of the membrane interface and might involve interactions with other viral structures. Here we present results indicating that oligomeric complexes established between MPER and the conserved amino-terminal fusion peptide (FP) can partition into lipid vesicles and be specifically bound by the 2F5 antibody at their surfaces. Cryo-transmission electron microscopy of liposomes doped with MPER:FP peptide mixtures provided the structural grounds for complex recognition by antibody at lipid bilayer surfaces. Supporting the immunogenicity of the membrane-bound complex, these MPER:FP peptide-vesicle formulations could trigger cross-reactive anti-MPER antibodies in rabbits. Thus, our observations suggest that contacts with N-terminal regions of gp41 may stabilize the 2F5 epitope as a membrane-surface antigen.

## Introduction

Eliciting broadly neutralizing antibodies (NAbs) to human immunodeficiency virus type-1 (HIV-1) before infection becomes established, is one of the main objectives pursued in HIV vaccine design [1], [2]. Monoclonal antibody (MAb) 2F5 is among the antibodies with the broadest heterologous HIV-1 neutralizing activity and has been shown to protect against viral infection when passively transferred to primate models [3], [4]. MAb2F5 recognizes a linear epitope sequence within the conserved membrane-proximal external region (MPER) of the fusogenic Env subunit gp41 [5]–[8]. MPER is however poorly immunogenic in the context of the viral infection or upon immunization with Env-derived subunit vaccines and, therefore, constitutes a timely candidate for development of a peptide-based vaccine (for comprehensive reviews see [9]–[11]).

**Figure 1:**
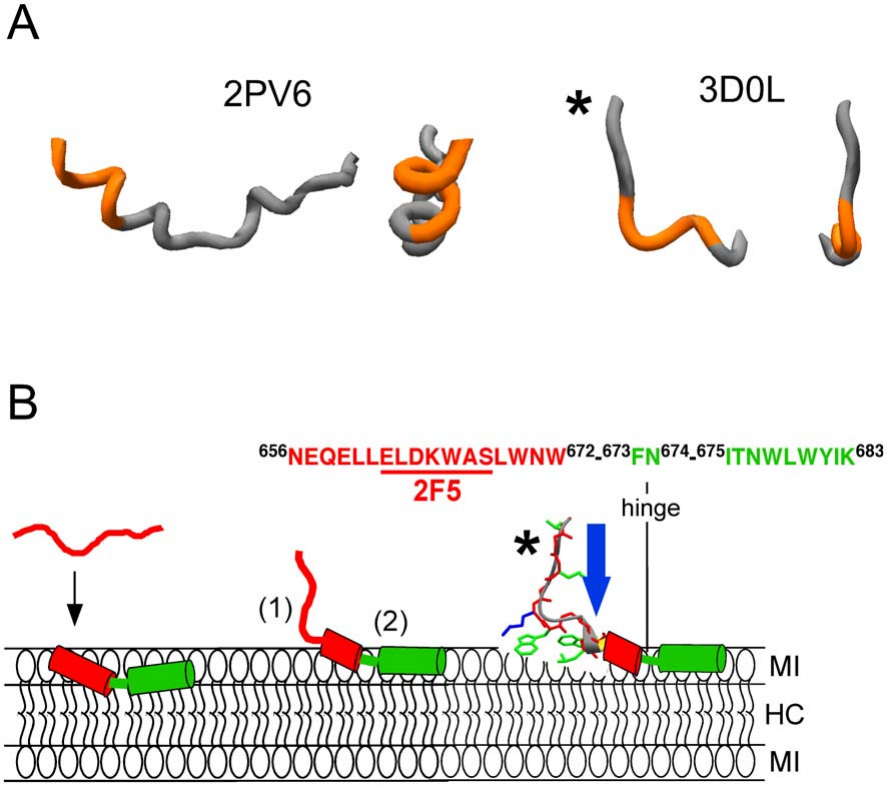
Proposed organization for the 2F5 epitope at membrane interfaces. A) Secondary structures adopted by the ^662^ELDKWAS^668^ sequence spanning the 2F5 core epitope (depicted in orange), in contact with DPC micelles (left) or in complex with Fab’2F5 (right). Lateral and front views of the structures (PDB accession numbers as indicated in the panel) were rendered using Swiss-PDB-viewer program. B) MPER sequence (top) and membrane topologies proposed for the 2F5 epitope (bottom). Bottom-left: Membrane-inserted MPER peptide. Partitioning from solution constrains 2F5 epitope into a helical structure mostly embedded into the hydrocarbon core (HC) region of the bilayer (red cylinder) [30]. Bottom-center: Hypothetical MPER structure recognized by MAb2F5 in the vicinity of the membrane interface (MI). (1) and (2) respectively indicate the structure bound to the high-affinity binding site and the surface putatively contacted by the hydrophobic loop [38], [39]. Bottom-right: Proposed FP effect on 2F5 epitope stabilization. FP residues (blue arrow) may retain the 2F5 epitope β-turn structure at membrane interfaces [27] and orient downstream residues for additional antibody docking. The 2F5 epitope region has been depicted as in the structure with PDB entry 3D0L (asterisk). Green side-chains designate hydrophobic residues.

In accordance with the existence of distinct structural MPER states during Env biosynthesis, virion assembly, and membrane fusion [12]–[17], peptides representing the linear 2F5 epitope sequence display conformational flexibility [18]–[24]. However, crystal structures of the antigen-binding fragment (2F5 Fab') complexed with peptide have revealed a well-defined type I β-turn conformation for the ^662^ELDKWAS^668^ core-epitope sequence [25]–[27], consistent with the existence of a defined 2F5 target structure within gp41. Recent crystallographic analysis by Bryson et al. [28] supports that both, proper epitope β-turn conformation, and side-chain positions are crucial for efficient 2F5 binding and broad neutralization. Explicitly, within ^664^DKW^666^ residues, which are key for 2F5 recognition, Asp-664’s negative charge position and alkyl-π stacking between Lys-665 and Trp-666 side-chains must be preserved. An amino-terminal extended stretch comprising residues ^656^NEQELL^661^ is additionally observed in peptide epitopes elongated to increase antibody affinity [8], [26], [27]. It is therefore likely that the extended+β-turn kinked structure recognized by 2F5 may be structurally fixed through tertiary interactions with other viral structures in the gp41 native state (Fig. 1). If so, recreating such an arrangement could be crucial for eliciting 2F5-like antibodies through vaccination.

The proximity to the envelope surface and the distribution of polar and non-polar (aromatic and aliphatic) residues further suggest that among the various MPER states a low-energy structure may exist inserted into the viral membrane external interface [12], [29], [30] (Fig. 1). Accordingly, water-soluble and disordered 2F5 epitope-representing ^656^NEQELLELDKWASLWN^671^ peptide (2F5ep in Table 1) may become membrane-bound and structured upon addition of the C-terminal, aromatic-rich stretch rendering it the full ^656^NEQELLELDKWASLWNWFNITNWLWYIK^683^ MPER sequence (MPERp in Table 1) [31]. Anti-MPER 2F5, Z13e and 4E10 antibodies can bind to epitopes buried in the membrane, and are thought to induce their partial extraction [30]–[34]. Thus, MPER structurally constrained through membrane partitioning-coupled folding might in principle encompass a relevant anti-MPER immunogen, i.e., with the potential of generating 2F5-like, neutralizing antibodies. However, the NMR structure of an MPER-based peptide embedded in dodecylphosphocholine (DPC) micelles displays a well-defined α-helical conformation for the 2F5 core epitope ELDKWAS sequence [30] (Fig. 1A-right). This equilibrium conformation anticipates the observed energy penalty for membrane-inserted 2F5 epitope binding [31], [34], as well as the failure in eliciting 2F5-like antibodies through vaccination with liposome-peptide formulations [16], [35].

**Table 1:** Peptide sequences used in this study (core 2F5 epitope residues underlined).

Name	Sequence	Gp160 numbering^a^
**FPp**	AVGIGALFLGFLGAAGSTMGARSKKK	512–534-KKK
**FPctl**	AFVTLSGARGAGAGSGMLILGAFKKK	512–534scr-KKK
**FPp-Cys17**	AVGIGALFLGFLGAAG**C**TMGARSKKK	512–534(S528C)-KKK
**C34**	WMEWDREINNYTSLIHSLIEESQNQQEKNEQELL	628–661
**2F5ep**	NEQELLELDKWASLWN	656–671
**2F5ep-Cys**	NEQELLELDKWASLWNC	656–671-C
**MPERp**	NEQELLELDKWASLWNWFNITNWLWYIK	656–683
**MPERp(9,10)Ala**	NEQELLEL**AA**WASLWNWFNITNWLWYIK	656–683
**preTM**	DKWASLWNWFNITNWLWYIK	664–683
**CpreTM**	KKKNWFDITNWLWYIKLFIMIVGGLVKK	KKK-671–693-KK
**TMDp**	KKKLFIMIVGGLVGLRIVFAVLSIKKK	KK-684–704-KKK

a sequences and numbering are derived from the prototypic HXBc2 isolate.

In this work we assume that MAb2F5 belongs to a class of neutralizing antibodies that prevent infection by diverse human viruses following a common mechanism: tight binding to conserved structures within envelope glycoprotein stem regions and subsequent blocking of membrane fusion [36]. Previous work by our group suggested that amino-terminal gp41 FP residues might stabilize 2F5 core-epitope non-helical structure [22], [23], [27], [37]. More recently it has been suggested that the solvent-exposed section of the downstream MPER helix might constitute an additional docking surface for the antibody [38], [39]. We test here the hypothesis that the MPER sequence may be constrained into a relevant 2F5 epitope-mimic at membrane surfaces through simultaneous interactions with the N-terminal FP sequence and the membrane interface (Fig. 1B). Our data indicate that MPER/FP peptide complexes formed in solution, can partition from water into lipid bilayers, be preserved within the low-moderate polarity membrane-interface environment, and be distinctly recognized by the MAb2F5 on membrane surfaces. Moreover, vesicles that contained MPER/FP peptide mixtures were immunogenic in rabbits when administered together with muramyl dipeptide (MDP) adjuvant, and induced antibodies that targeted the 2F5 epitope region. Altogether, these observations support the idea that the FP residues may stabilize 2F5 epitope at the membrane surface for effective MAb2F5 and B-cell receptor binding.

## Results

### MPERp/FPp Complex Formation in Solution

**Figure 2:**
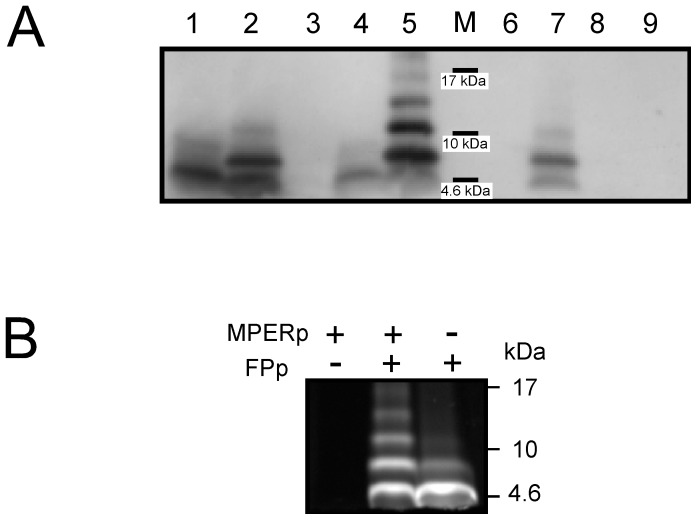
Electrophoretic analyses of MPERp/FPp complex formation. A) Peptides (10 µg) were processed by SDS-PAGE, and subsequently detected by Western blotting using 2F5 antibody. In some instances, peptides were pre-incubated with cross-linker (20 µM BS^3+^). Samples were loaded onto the gel as follows: (1) MPERp; (2) MPERp/BS^3+^; (3) MPERp(9,10)Ala/BS^3+^; (4) MPERp:FPp (1∶1) mixture; (5) MPERp:FPp/BS^3+^; (6) MPERp(9,10)Ala:FPp/BS^3+^; (7) MPERp:FPctl/BS^3+^; (8) FPp/BS^3+^; (9) FPctl/BS^3+^. M: positions of the molecular weight markers (MW: 17, 10 and 4.6 kDa). B) Fluorescence detection of NBD-labeled FPp in SDS-PAGE gels. All samples were incubated with 20 µM BS^3+^ before electrophoresis. The migration of molecular weight standard proteins is indicated in the frame.

We first performed a combined cross-linking and Western blot analysis to assay complex formation by the FP (FPp) and MPER (MPERp) peptides. Results displayed in Fig. 2A demonstrates the induction of higher order MPERp complexes by the FP sequence in solution. The 2F5 antibody bound to MPERp monomers (lane 1) and to dimers (lane 2) generated after incubation of the peptide with BS^3+^ cross-linking agent in solution prior to SDS-PAGE. Specific recognition in these assays was supported by the absence of antibody binding to the MPERp(9,10)Ala peptide (lane 3). In this cognate MPER peptide, the key recognition pattern, ^664^Asp-Lys^665^_,_ was substituted for the Ala-Ala dipeptide [40]. Upon incubation of MPERp with the FPp, higher sized, cross-linked products were bound by the antibody (compare lanes 4 and 5). Again, recognition was not detected for cross-linked MPERp(9,10)Ala/FPp mixtures (lane 6). The specificity of the cross-linking reaction was further assessed using FPctl, a fusion peptide variant with same amino acid composition but scrambled residues (lane 7). Upon incubation with BS^3^, MPERp/FPctl and MPERp alone samples displayed similar band patterns (compare lanes 7 and 2, see also below). Thus, the FPctl peptide could not reproduce FPp-induced oligmerization, indicating that the observed increase in cross-linking was depending on the wild-type FP sequence and not merely due to its overall hydrophobicity. Finally, FPp or FPctl were not directly recognized by the antibody under those conditions (lanes 8 and 9).

To test whether FPp also underwent oligomerization upon incubation with MPERp, experiments were carried out using NBD-labeled FPp, which retained the capacity of the unlabeled peptide for inducing MPERp cross-linking (see below), while allowing detection in gels through NBD fluorescence emission (Fig. 2B). The appearance of higher MW peptide bands in those assays indicates that incubation with MPERp also elicited efficient FPp cross-linking.

Under the conditions of the SDS-PAGE and WB experiments different degrees of MAb2F5 reactivity were observed (e.g., compare lanes 2 and 4). To determine whether the MPER/FP peptide complex was better recognized by MAb2F5 than MPERp alone, we carried out a dot-blot assay (Fig. S1, left panel). The data confirmed better MAb binding to MPERp:FPp mixtures than to MPERp alone samples.

### MPERp/FPp Complex Incorporation into Membranes

**Figure 3:**
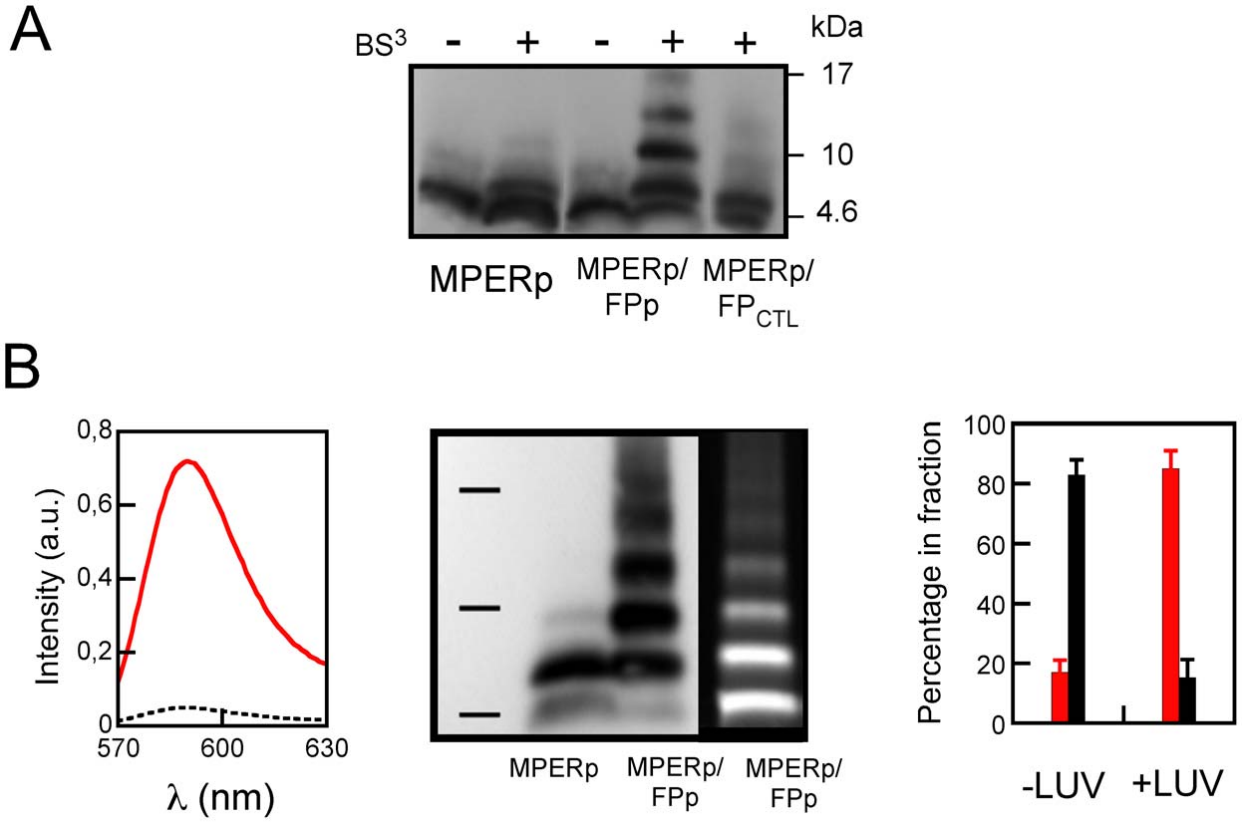
Preservation of MPER/FP complexes upon association with membranes. A) Western blot analyses of MPER-based peptides pre-incubated with lipid vesicles at a peptide-to-lipid ratio of 1∶50, prior to cross-linking with 20 µM BS^3+^ and electrophoresis. Conditions otherwise as in previous Fig. 2A. B) Evaluation of complex-membrane association by vesicle floatation. Peptide-containing vesicles (added peptide-to-lipid ratio of 1∶50) were treated with 20 µM BS^3+^ cross-linker and then subjected to ultracentrifugation (627,000×g) 120 min in D_2_O buffer. Left panel: Fluorescence emission of Rhodamine-labeled POPC:Chol (2∶1) LUV recovered from floating (red-solid) and non-floating (black-dotted) fractions. Center Panel: MPERp and FPp co-floating with vesicles were detected by Western blotting with MAb2F5 or following NBD fluorescence, respectively. Right panel: Percentage of peptide in floating and non-floating fractions (red and black bars, respectively) in the presence and absence of vesicles (LUVs).

In combination, results displayed in Fig. 2 were consistent with the formation of specific MPERp/FPp complexes that allowed effective cross-linking of constituent monomers. By comparison, cross-linking efficiency for the respective sequences alone was greatly reduced. The fact that these complexes were not SDS-resistant suggested that they might disassemble in contact with hydrophobic environments. Thus, we next sought to establish whether insertion into lipid bilayers might solubilize MPERp/FPp complexes formed in solution. Results displayed in Fig. 3A correspond to peptides pre-incubated with lipid vesicles and subsequently treated with the cross-linking agent in some instances. The data reveal that FPp-dependent cross-linking efficiency was not reduced by pre-incubation with the lipid vesicles.

To evaluate the amount of complexes formed in solution that actually bound to membranes, liposomes were incubated with the peptides and subsequently floated in D_2_O buffer by means of ultracentrifugation [41]. In these experiments, floatation of the vesicles was followed by the fluorescence of Rhodamine-labeled phospholipid incorporated into the lipid composition (Fig. 3B-left). The upper volume, which after centrifugation contained ca. 95% of the vesicles, was defined as the floating fraction. Vesicle-bound MPERp and NBD-FPp present in this fraction were detected by Western blot and fluorescence emission, respectively (Fig. 3B-center). Effective MPERp/FPp cross-linking could be observed in the peptide mixtures co-floating with vesicles, thereby confirming that complexes were retained in contact with vesicle surfaces. In the MPERp/FPp mixture samples, vesicle-bound/solution-free peptide ratios ranged between 8/2 and 7/3, as inferred for both MPERp monomers and cross-linked species (Fig. 3B-right). From these experimental data an approximate water-membrane mole-fraction partitioning coefficient (*K*_x_) of 6.5±1.6×10^5^ could be estimated, which was subsequently used for determining the amount of membrane-bound complex under different experimental conditions.

### MAb2F5 Binding to Liposomal MPERp/FPp Complexes

**Figure 4:**
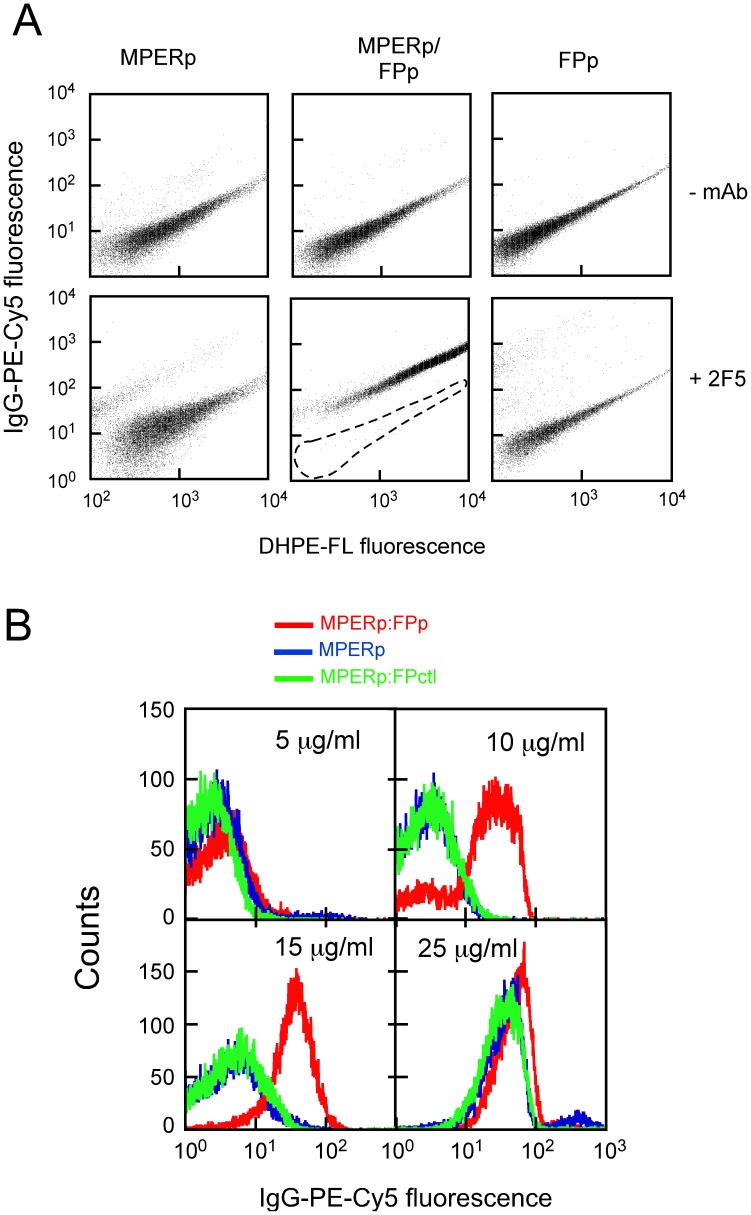
MAb-vesicle association as determined by flow cytometry. A) The MAb2F5 was incubated with a stirred solution of f-DHPE-labeled LUV (250 µM). After 10 minutes, the fluorescently labeled secondary Ab was added and the resulting mixtures were incubated for 5 minutes before being analyzed by flow cytometry. Top panels: the samples in the absence of MAb show a single particle population that corresponds to DHPE-labeled vesicles. Bottom panels: the incubation with 15 µg/ml of MAb rendered double labeled vesicles in MPERp:FPp mixture-containing samples (center), but not in MPERp- (left) or FPp (right) containing samples. This pattern could not be observed in the absence of the MAbs (top panels). B) Specificity of FPp effect on MAb2F5-vesicle association as detected by FACS. MAb2F5 was incubated at the final concentrations indicated in the panels with MPERp:FPp mixture-, MPERp- or MPERp:FPctl mixture-containing LUV (red, blue and green traces, respectively) under conditions that were otherwise similar to those in the previous panel.

**Figure 5:**
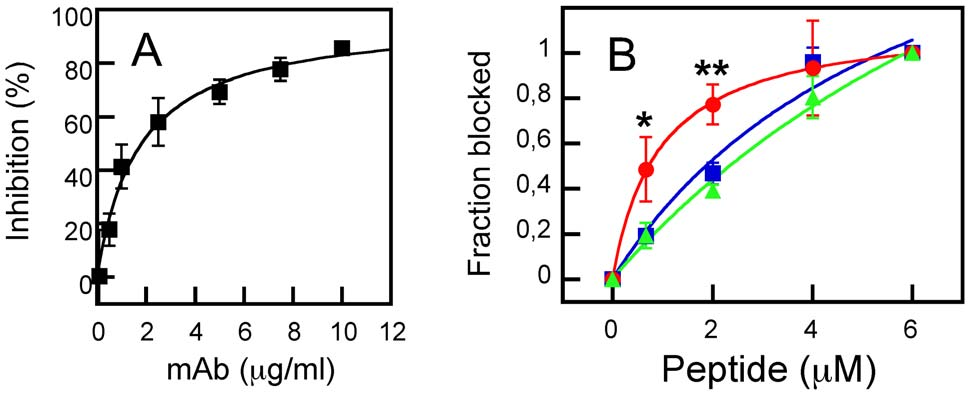
Reversion of MAb2F5-mediated fusion inhibition after pre-incubation with MPERp/FPp peptide complexes on LUV. A) MAb2F5 capacity for inhibiting gp41-mediated cell-cell fusion. B) Pre-incubation with LUVs containing the MPERp:FPp mixture (red circles), the MPERp only (blue squares), or the MPERp:FPctl mixture (green triangles) restores gp41-mediated cell-cell fusion that is otherwise inhibited by MAb2F5. MAb was applied at 5 µg/well in these assays. Lipid and membrane-bound peptide concentrations were 500 and 4.5 µM, respectively. Significant differences are indicated (**p<0.005; *p<0.05). Means ± SD of four experimental determinations are displayed in both panels.

**Figure 6:**
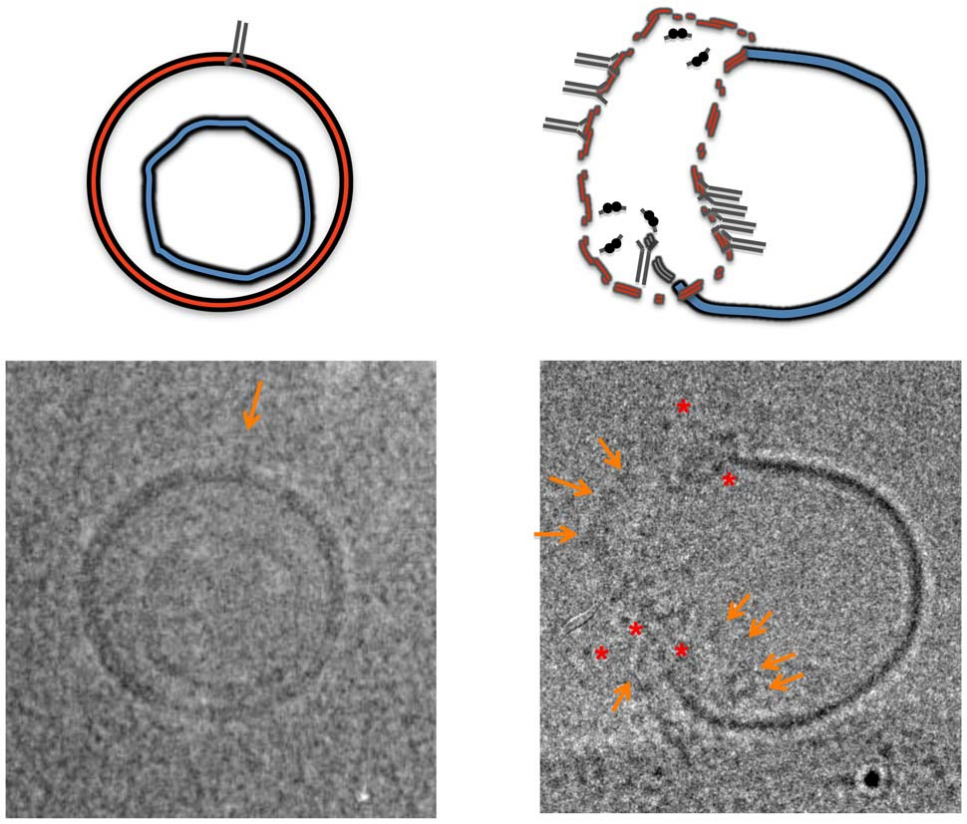
MAb2F5 recognition of membrane-bound MPERp/FPp complex by cryo-TEM. Micrographs correspond to LUV pre-incubated with MPERp:FPp mixture (complex-to-lipid mole ratio of 1∶100), that were subsequently incubated for 20 seconds with MAb2F5 (paratope-to-peptide ratio of 1∶10) before vitrification (left panel), or vitrified after 5 minutes of incubation with double concentration of MAb (paratope-to-peptide ratio, 1∶5) (right panel). Arrows point to rods of approximately 10–12 nm that protrude from the bilayer surface, while asterisks indicate electrodense particles. Both vesicles are shown at the same magnification. Cartoons on top depict the interpretation for the observed structures (see text). In the right micrograph, gold particle’s diameter was 10 nm (bottom right corner).

Overall, the previous results suggest that MPERp/FPp complexes partition into vesicles and retain a specific structure, which allows their cross-linking also on membrane surfaces. Dot-blot experiments further suggested that MAb2F5 bound to MPERp:FPp mixtures more efficiently than to MPER alone also in the presence of lipids (Fig. S1, right panel). Moreover, antibody binding was more efficient in peptide-lipid mixtures than in peptide-alone samples (compare right and left panels). These observations support a role for the FP and the membrane in stabilizing the 2F5 epitope (Fig. 1B). However, variable degrees of lipid hydration, induction of bilayer mechanical stress and/or lipid packing defects make lipids deposited on solid supports poorly defined membrane systems. Therefore, to determine degrees of 2F5 epitope accessibility within membrane-bound structures, antibody binding was determined in solution-dispersed large unilamellar vesicles (Figs 4–6).

A flow-cytometry assay was first used to simultaneously detect liposome and antibody fluorescent signals [31]. As shown in Fig. 4A, upon incubation with 15 µg/ml MAb2F5, an antibody-stained vesicle population could only be recovered from samples containing MPERp:FPp mixture and MAb2F5 (center-bottom panel). Samples containing only MPERp (left-bottom) or FPp (right-bottom) showed the fluorescence signal corresponding to control samples devoid of MAb (top panels). These results would be consistent with more efficient 2F5 epitope presentation on membrane surfaces in the presence of the FPp sequence. Moreover, they emphasize the absence of spontaneous MAb-membrane association under these conditions.

In the experiments displayed in Fig. 4B, vesicles were titrated in solution with increasing amounts of antibody. The histograms reveal that vesicles labeled with fluorescent antibody were already recovered from MPERp:FPp mixture-containing samples incubated with 10 or 15 µg/ml of MAb2F5 (red traces). At those antibody doses MPER-containing vesicles were not stained by the fluorescent IgG (blue traces), while incubation with 25 µg/ml of MAb2F5 stained the vesicles independently of the FP. Vesicle-labeling pattern as a function of the MAb2F5 dose was actually comparable in MPER alone and MPERp:FPctl mixtures (green traces), confirming that the scrambled sequence did not mimic the FPp stimulatory effect on binding.

Titration in solution of MAb2F5 with increasing concentrations of peptide-containing vesicles supported the previous observations (Fig. 5). In those assays antibody-membrane partitioning was inferred from the capacity of the vesicles for competing with binding to native gp41. Neutralizing 2F5 antibody recognizes native gp41 expressed on cell surfaces and inhibits its cell-cell fusion activity [42]. Accordingly, MAb2F5 incubation with Env-expressing cells results in the inhibition of their fusion with CD4-expressing cells in a dose-dependent manner (Fig. 5A). Thus, the degree of native 2F5 epitope structural mimicry by the membrane-associated MPERp/FPp, can be inferred from the capacity of liposome-peptide complexes to interfere with MAb2F5 binding to functional gp41 expressed on the cell surface (Fig. 5B). All the peptide-containing vesicles reversed the MAb2F5-induced blockage of syncytium formation, while vesicles devoid of peptide had no effect (not shown). The dose dependency of the inhibitory effect confirmed that MPERp/FPp mixture-containing vesicles (circles and red line) were significantly more efficient formulations than those containing MPERp alone (squares and blue line) or MPERp/FPctl mixtures (triangles and green line). Hence, these data further substantiate a better structural mimicry of 2F5 antigen by the FPp-stabilized complexes.

### Ultrastructure of MAb2F5 Bound to Liposomes

Cryo-transmission electron microscopy (cryo-TEM) analyses supported MAb2F5 binding to vesicle surfaces containing MPERp/FPp complexes (Figs. 6, S2 and S3). Vitrified samples of LUV doped with MPERp/FPp mixtures and incubated for 20 s with antibody (Fig. 6-left and S2), disclosed double-rod structures (arrows), consistent with single IgG-s protruding from the membrane surrounding the liposomes (depicted in gray in the top diagram). The stomatocyte-like morphology of most vesicles, typical of LUV produced through extrusion [43], was unaltered under these conditions. In contrast, incubation of these vesicles for longer times with higher doses of antibody had remarkable effects on vesicle morphology and membrane surface appearance (Fig. 6-right and S3).

First, the majority of the vesicles treated with high antibody doses formed clusters (Fig. S3A), most likely due to inter-vesicle bridging by the bivalent antibodies. In addition, many vesicles lost the stomatocyte-like morphology and showed a membrane-evagination pattern [43]. In those vesicles two regions were clearly discernable (Fig. 6-right and S3B). The evaginated membrane (indicated in blue) was devoid of antibody particles. By comparison, the initially accessible membrane region (depicted in red) was crowded with antibodies. In the antibody-containing areas, palisades of sticking out double-rods (arrows) combined with electrodense particles (asterisks) and the lipid bilayer could be barely discerned (dotted lines, see also Figs. S3 C–F). We infer that these structures correspond to lateral and top views of the membrane-associated antibodies, respectively (grey rods and black dots in the explanatory cartoons of Fig. 6). It has been shown that increasing the external monolayer surface area causes loss of LUV invaginations [43], [44]. Thus, we surmise that MAb binding to epitope results in mass addition to the membrane external leaflet.

### Immunogenicity of Peptide-lipid Formulations

**Figure 7:**
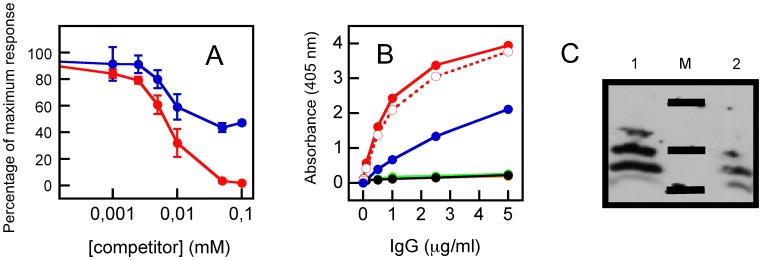
Recovery of 2F5 epitope targeting antibodies from rabbit serum. A) Competitive ELISA assays were performed using plates coated with MPERp/FPp (1.4 µM). Prior to adding to the plates, 10 µg/ml of total (blue) or 0.1 µg/ml of 2F5-specific (red) IgG purified from rabbit sera was pre-incubated for 30 minutes with serial dilutions of 2F5ep competitor and set-up in triplicates. B) Peptide recognition by rabbit IgG purified with 2F5ep-Cys. Antibodies were titrated against 1.4 µM of the following peptides immobilized in ELISA plates: C34 (orange); MPERp (red); CpreTM (green); TMDp (black). Reactivity to 2F5ep is denoted by empty red symbols and dotted line. Specificity of the response for the correct 2F5 epitope was tested using MPERp(9,10)Ala (blue). C) Reactivity with liposome-peptide immunogen by Western blot. Liposome-associated peptides were incubated with 20 µM BS^3+^ as described in the caption for Fig. 3A, subsequently resolved by SDS-PAGE electrophoresis, then transferred to a nitrocellulose membrane and finally probed with 2F5ep-specific antibodies. Lanes 1 and 2 display bands corresponding to MPERp/FPp and MPERp(9,10)Ala/FPp mixture samples, respectively. M: molecular weight markers (MW: 17, 10 and 4.6 kDa).

The MAb binding pattern described in the previous sections warranted the assessment of vesicle-bound MPERp/FPp capacity for activating B-cell responses. Thus, rabbits were immunized with liposome-peptide formulations prepared under conditions that ensured total incorporation of the peptide complexes into vesicles. Antigen-specific antibodies were recovered upon immunization with the MPERp/FPp equimolar mixture associated to lipid vesicles (Figs. 7 and S4). Cross-reactivity assays confirmed that sera recovered from rabbits immunized with complex-containing liposomes contained antibodies that recognized MPERp, the 2F5 lineal epitope-representing peptide (2F5ep, Table 1) and recombinant gp41 (Fig. S4). However, no-antibodies were raised against the C34 or preTM peptides, encompassing residues N- or C-terminal to the core 2F5 epitope, respectively (Table 1).

To further assess the level of 2F5-like responses induced by the membrane-associated MPER/FP complex, we isolated IgGs specific for the 2F5 epitope from sera, and compared them with the total IgG fraction in competitive ELISA (Fig. 7A). Soluble 2F5ep peptide epitope inhibited completely specific IgG binding to MPERp/FPp mixtures deposited on plates (red symbols). In contrast, inhibition by this peptide of the total IgG fraction binding was partial (blue symbols). The 2F5-specific IgG-s bound to MPERp and 2F5ep in ELISA, but not to peptides representing N- or C-terminal gp41 sequences devoid of the 2F5 core epitope residues (Fig. 7B and Table 1). However, in specificity controls these IgGs also reacted to a certain extent with MPERp(9,10)Ala, a peptide with 2F5 epitope key residues substituted by Ala (Fig. 7B and Table 1). The WB analysis displayed in Fig. 7C confirmed recognition of liposome-associated MPERp/FPp complexes by the 2F5ep-specific IgG-s, but again, the IgG-s also reacted with liposomes loaded with MPERp(9,10)Ala/FPp complexes.

Finally, 2F5-specific IgG-s purified from sera were tested for inhibition of infection by homologous virus. In neutralization assays employing AA-2 cells and infectious IIIB viruses, only a weak neutralizing activity (IC_50_ 22.5 µg/ml) could be detected for anti-2F5ep IgGs isolated from the MPERp:FPp sera. For comparison, MAb2F5, included as a positive control, displayed an IC_50_ value of 0.11 µg/ml in these assays.

## Discussion

Systematic analyses of sera collected from chronically infected individuals have revealed that some HIV-1 neutralizing activity can be mapped to the gp41 MPER domain [11], [45]–[50]. These observations confirm that this conserved Env sequence can be immunogenic during infection. However, production of anti-MPER vaccines is currently limited by the poor understanding of the viral and/or host factors underlying efficient development of antibodies targeting this region [11]. Based on cross-reactivity with lipids of anti-MPER antibodies, some authors argue that B cells producing 2F5-like antibodies are self reactive and therefore deleted during differentiation [51]. Contrasting this view, other studies have failed to establish a relationship between 2F5 lipid polyreactivity and the requirement of self-tolerance break for development of cognate anti-MPER antibodies [52]–[55]. Notably, under our experimental conditions, we did not detect any direct interaction between MAb2F5 and lipid vesicles devoid of peptide-epitope. We have recently argued that the direct association of MAb2F5 with bilayers made of anionic phospholipids is driven by electrostatic rather than hydrophobic interactions [35]. In line with this idea, dot-blot, flow cytometry, reversion of fusion inhibition, and cryo-TEM results concurred in showing absence of MAb2F5 interaction with electrically neutral membranes composed of PC and Chol (Figs. 4–6 and S1).

In addition, claims have been made that high levels of somatic hypermutation are required for broadly neutralizing antibodies to evolve, and that such requirement would pose a challenge for elicitation of MPER antibodies through vaccination [46], [56], [57]. However, recent identification of the monoclonal antibody m66.6 has demonstrated that cross-neutralizing antibodies with 2F5-like specificity, but lower level of somatic hypermutation, can indeed be elicited during infection [46]. Recent reports on the recovery of modest amounts of anti-MPER neutralizing antibodies through vaccination [50], [58], also suggest that conditions of persistent infection might not be absolutely required for eliciting them [57].

An alternative, non-exclusive option establishes that development of effective anti-MPER vaccines is to date hampered by our limited knowledge on the native gp41 structures competent in raising neutralizing antibodies [59]. Identification and structural mimicry of those structures would be required to recover neutralizing responses through vaccination [60]. Particularly, the viral structures recognized by 2F5 antibody to exert neutralization may depend on defined conformations of the lineal epitope, tertiary interactions with other Env regions, transient accessibility and/or interactions with membrane lipids [23], [26]–[28], [30], [34], [35], [37], [59], [61]–[65].

In the search for structure-based immunogens targeting 2F5, initial strategies focused on conformational stabilization of the core epitope. For instance, the ELDKWAS sequence was conformationally constrained either through cyclation of synthetic peptides [66], [67], or by grafting it into recombinant protein loops [68]–[70]. These constructs were bound with higher affinity by MAb2F5, but incapable of triggering relevant neutralizing responses, most likely because they did not reproduce the structural requirements for eliciting 2F5-like antibodies [28]. More recently, following a rational “scaffolding” approach, the ^659^ELLELDKWASL^669^ sequence has been transplanted into proteins exposing peptide chains that assume the conformation of the Fab-bound 2F5 epitope [71], [72]. Crystal structures of these epitope-scaffolds (ES) in complex with the antibody confirmed the adoption of the standard epitope conformation by the grafted sequence. Nonetheless, antibodies recovered after ES immunization did not neutralize the virus. Crystallographic analyses of the 2F5 peptide epitope in complex with monoclonal antibodies elicited by the ES provided a likely structural explanation for the lack of neutralizing activity [60], [71]. Peptide-epitopes in complex with Fab2F5 or ES-elicited monoclonals adopted comparable conformations and oriented side-chains in remarkably similar ways. However, ES-elicited antibodies lacked a hydrophobic, long CDR-H3 loop, which is essential for Fab2F5’s neutralizing activity [38], [64], [65], [73]. It has been argued that, in addition to specific interactions of the high-affinity binding site with the core epitope sequence, interactions of the loop with the viral membrane and/or downstream MPER regions are required for accomplishing the neutralizing activity of MPER-targeting antibodies [34], [38], [39], [64], [65], [74], [75] (Fig. 1B-center).

Our previous work suggested that 2F5 epitope structure could be stabilized trough specific interactions with the gp41 FP sequence [23], [27]. Here, we hypothesize that complexes formed as a consequence of those interactions might embody 2F5-like relevant structures when adsorbed to the membrane interface (Fig. 1B-right). Thus, the experimental work presented here was designed to test whether membrane-bound MPERp/FPp complexes could fulfill the requirements for eliciting 2F5-like antibodies, namely, i) retention of 2F5 epitope residues that contribute to high-affinity binding in a relevant conformation, and ii) presentation of such structure in the natural context provided by the membrane surface, i.e., potentially accessible to antibodies with long, hydrophobic CDR-H3 loops.

### 2F5 Epitope Recognition in Membrane-bound MPERp:FPp Mixtures

The increased binding of MAb2F5 to vesicles containing MPERp/FPp, as compared to MPERp only-containing vesicles (Figs. 4, 5 and S1), supports that MPERp/FPp complexes displayed on membrane surfaces retained the 2F5 epitope in a more relevant conformation. In previous work we reported that the FP sequence could constrain the 2F5 epitope into non-helical conformations in moderate-low polarity media [22], [23]. These non-helical conformations underwent a transition to helical structures upon further decrease of the medium polarity [37]. A moderate decrease of the medium polarity also accompanies the peptide partitioning from the water phase into the membrane interface, while deeper insertion into the hydrocarbon core correlates with lower polarity (Fig. 1B-left). Structural data displayed in Fig. S5A indicate that the MPERp, when co-mixed with FPp, actually adopted in vesicles and the moderate-low polarity medium provided by 5% 1,1,1,3,3,3-hexafluoro-2-propanol (HFIP) similar structures, which were previously shown to be consistent with an increase in β-turn conformer population [22]. Those results suggest that in the moderate-low polarity media provided by membrane surfaces or 5% HFIP the structures distinctly adopted by MPERp:FPp mixtures were preserved.

To examine the effect of lower polarity on these complexes, the structural characterization was also carried out in the presence of the dodecylphosphocholine (DPC) detergent or 25% HFIP (Fig. S5B). DPC has been the membrane-mimetic of choice for solution NMR experiments involving sequences similar to the ones employed in our study [30], [76]. Consistent with the NMR data, CD analyses of the MPERp:FPp mixture samples treated with the detergent disclosed spectra compatible with the adoption of main helical structures (Fig. S5B, left panel). These measurements did not reveal interactions between MPERp and FPp. Moreover, in DPC micelles or low-polarity 25% HFIP the peptides adopted comparable conformations (Fig. S5B, right panel). The reduction of inter-peptide interactions in the DPC samples was additionally supported by cross-linking results (Fig. S5C). Thus, incubation with DPC prior to cross-linking resulted in the disappearing of the MPERp and FPp oligomeric species, which were otherwise observed when cross-linking was carried out in the presence of vesicles (right and left panels, respectively). Taken together these results suggest that MPERp and FPp would disassemble into non-interacting helices upon insertion into more hydrophobic environments of the lipid bilayer.

### Immunogenicity of Membrane-bound MPERp:FPp Mixtures

Overall, the previously discussed results suggest that the membrane interface might provide the suitable environment for 2F5 recognition within MPERp/FPp complexes. To probe whether the membrane-bound complexes can indeed interact with B-cell receptors and activate an anti-MPER humoral response, we evaluated the immunogenicity of complex-containing liposomes in rabbits. Data displayed in Figs. 7, S4 and S6 demonstrate that peptide mixture-containing vesicles could trigger an MPER-targeting response. The antibodies elicited upon immunization with MPERp/FPp-containing vesicles were cross-reactive, and recognized the liposome-associated peptide complexes by Western blot following a MAb2F5-like pattern (Fig. 7). However, in contrast to the MAb, antibodies recovered from rabbit sera also bound to the peptide-epitope with key recognition residues ^664^Asp-Lys^665^ substituted for by Ala-Ala [28], [40].

We have recently reported that MPERp attached to vesicles made of anionic phospholipids also triggers a comparable immunogenic response [35]. However, 2F5ep-specific IgGs isolated from those sera were devoid of any neutralizing activity at the highest tested concentrations (i.e., 200 µg/ml). In contrast, we observe here neutralizing activity above 25 µg/ml for the IgGs isolated in the same way. This might be consistent with a higher degree of epitope mimicry by the MPERp/FPp-vesicle formulations used in this study. If this were the case, binding to complex structures outside the 2F5 epitope might also result in viral neutralization. Competitive ELISA actually revealed that soluble 2F5ep could not fully inhibit binding of the total IgG fraction to complexes deposited on plates (Fig. 7A), suggesting the induction by complexes on vesicles of antibodies different from Mab2F5.

To determine the functional characteristics of such antibodies, we attempted their purification from rabbit immune sera with the MPER/FP-vesicle complexes immobilized in affinity columns. To that end we synthesized an FPp-based sequence with a Ser × Cys substitution at the C-terminal side (FPp-Cys17) (see Table 1). Quantification of lipid and peptide revealed that MPERp/FPp-Cys17-vesicle complexes could be immobilized in columns, albeit with lower yields than 2F5ep-Cys. Even though, specific IgG could be isolated in sufficient amounts as to perform the epitope-mapping and neutralization experiments displayed in Fig. S6. IgGs specific for complexes displayed on the surface of vesicles bound to residues that roughly span the extended 2F5 epitope, and also to the FP (Fig. S6A). Thus, the IgG fraction different from 2F5 (i.e., not inhibited by soluble peptide epitope in Fig. 7A), seems to target the FP. Pseudovirus-based neutralizing assays revealed that the complex-specific IgGs did not show improved neutralization efficiency as compared to those isolated from 2F5ep-Cys columns (Fig. S6B). We surmise that essentially similar 2F5 epitope-targeting neutralizing antibodies were isolated in MPERp/FPp-Cys17-vesicle and 2F5ep-Cys affinity columns.

In our assays, the neutralizing activity of antibodies recovered after affinity-purification was ca. 200 times lower than that displayed by the MAb2F5. One possible explanation for this difference is that a great deal of the polyclonal antibodies induced by MPER/FP complexes on vesicles bound slightly outside of the 2F5 key epitope region (see Fig. 7C). Recent attempts to recover MPER-targeting neutralizing antibodies through vaccination with membrane-based immunogens suggest that other factors can also affect the outcome of the response. Matyas et al. [63] described the production of neutralizing MAb-s with cross-reactivity qualitatively similar to 2F5, after immunizing mice with liposomes containing anionic phospholipid, MPER peptide and monophosphoryl lipid A as adjuvant. These MAb-s displayed neutralizing activity in the 10–100 µg/ml range of concentrations. MPER-targeting responses were also recovered by Ye et al. [58] from guinea pigs immunized with VLP-s displaying an influenza hemagglutinin-HIV-1 gp41 chimera. In this case recovered sera showed MPER-specific neutralization activity at 1/50 dilutions. Dennison et al. [16] reported that a recombinant-Env/MPER-liposome prime/boosting strategy elicited in rhesus macaques antibodies that specifically targeted to the 2F5 epitope. However, the recovered sera were devoid of neutralizing activity in this case. More recently, MPER constructs displayed on VLP surfaces were used by Kamdem Toukam et al. [77] to immunize mice. Again, the recovered sera contained 2F5 epitope-targeting antibodies but were devoid of neutralizing activity. In summary, the use of different adjuvants, immunization protocols, lipid compositions, or animal models may condition specificity and neutralizing activity of the recovered antibodies.

### Concluding Remarks

This work provides a proof-of-principle for the retention of an immunogenic MPER/FP complex at the surface of lipid vesicles. We surmise that further improvement of these liposome-peptide complexes to the level of potential 2F5-targeting vaccine candidates will require the optimization of the immunogenic formulations and vaccination protocols. It has recently been argued that priming with DNA expressing Env glycoproteins might be required to confer specificity to the immunogenic response [16], [58]. In this regard, our future research effort will focus on the improvement of the constituent peptides and lipids, the selection of effective adjuvants and the design of optimal prime-boosting protocols.

## Materials and Methods

### Materials

The MPER, TMD and FP-based sequences displayed in Table 1 were synthesized as C-terminal carboxamides by solid-phase synthesis using Fmoc chemistry, and they were purified by HPLC at the Proteomics Unit of the University Pompeu-Fabra (Barcelona, Spain). Peptide stock solutions were prepared in dimethylsulfoxide (DMSO, spectroscopy grade) and the concentrations were determined using a Bicinchoninic Acid microassay (Pierce, Rockford, IL, USA). The HIV-1 IIIB C34 peptide from DAIDS, NIAID was obtained through the NIH AIDS Research and Reference Reagent Program, Division of AIDS, NIAID, NIH. Neutralizing antibody-expressing hybridomas were originally generated by combined PEG-electrofusion of peripheral blood mononuclear cells of HIV infected non-symptomatic patients [78], and MAb2F5 used in this study was subsequently produced in recombinant CHO cells after the subclass switch to IgG1 [79]. 1-palmitoyl-2-oleoylphosphatidylcholine (POPC), phosphatidic acid (PA) and cholesterol (Chol) were purchased from Avanti Polar Lipids (Birmingham, AL, USA). Dodecylphosphocholine (DPC) was from Anatrace (Maumee, OH, USA). The N-(fluorescein-5-thiocarbamoyl)-1,2-dihexadecanoyl-sn-glycero-3-phosphoethanolamine (f-DHPE), fluorescent probe was from Molecular Probes (Junction City, OR, USA), and the PE-Cy5-conjugated mouse anti-human IgG was purchased from BD Biosciences. Recombinant gp41 from HXB2 molecular clone (*env* amino acids: 546–682) expressed in *Pichia pastoris* was obtained from the EU Programme EVA/MRC Centralized Facility for AIDS Reagents, NIBSC, UK (Grant number QLK2-CT-1999-00609 and GP828102).

### Production of Vesicles

Large unilamellar vesicles (LUV) were prepared following the extrusion method of Hope et al. [80]. Phospholipids and cholesterol were mixed in chloroform and dried under a N_2_ stream. Traces of organic solvent were removed by overnight vacuum pumping. Subsequently, the dried lipid films were dispersed in 5 mM Hepes, 100 mM NaCl (pH 7.4) buffer, and subjected to 10 freeze-thaw cycles prior to extrusion 10 times through 2 stacked polycarbonate membranes (Nuclepore, Inc., Pleasanton, CA, USA). The size distributions of the vesicles were determined using a Malvern Zeta-Sizer Nano ZS instrument (Malvern Instruments, Malvern, UK). Extrusion through membranes with a nominal pore-size of 0.1 and 0.2 µm produced POPC:Chol (2∶1 mol:mol) LUV with mean diameters (± S.D.) of 90±17 and 132±36 nm respectively. Lipid concentrations of liposome suspensions were determined by phosphate analysis.

### Peptide Analyses after Electrophoresis

For chemical cross-linking, 10 µg of peptide were incubated with 20 µM *Bis*[sulfosuccinimidyl]suberate (BS^3^) bifunctional cross-linking reagent (Thermo Fisher Scientific Inc.) for 1 h at 37°C. The reaction was stopped by adding Tris buffer (1 M, pH 7.5) to the mixture, as indicated by the manufacturer. Peptides were subsequently precipitated by trichloroacetic acid (TCA) and analyzed by 10–20% Tricine SDS-PAGE (NOVEX Tricine gels, Invitrogen). Peptides labeled with 4-nitrobenzo-2-oxa-1,3-diazole (NBD) fluorophore were visualized directly in gels using a VersaDoc Imaging System (BioRad). For Western blot, peptides were subsequently transferred to nitrocellulose membranes and probed with monoclonal antibody 2F5.

### Flow Cytometry

Antibody-vesicle association was determined using a BD FACScalibur Flow Cytometer (Becton Dickinson Immunocytometry Systems, Mountain View, CA) [31]. Measuring conditions were set for LUVs obtained through extrusion using filters with a nominal pore-size of 200 nm, a lipid concentration of 0.25 mM, and 0.01% fluorescent phospholipid probe (mole percent). Thus, in a typical experiment fluorescently labeled vesicles doped with peptide epitopes (1∶100 peptide:lipid molar ratio) were incubated for 10 minutes with increasing concentrations of the 2F5 MAb and subsequently, for 5 minutes with twice the concentration of anti-human immunoglobulin coupled to cyanine (IgG-PE-Cy5).

### Blockade of Syncytium Inhibition

MAb-peptide binding was evaluated in blockade of syncytium inhibition (or syncytium recovery) assays as previously described [23]. In brief, syncytium-formation assays were carried out using HXB2 *env* gene-expressing CHO-Env and CD4-expressing HeLaT4^+^ as effector and target cells, respectively (ARRRP-NIH, contributed by C. Weiss and J. White and R. Axel, respectively). Cell-cell fusion was inhibited by incubating CHO-Env cells with MAb2F5 for 90 min prior to co-culturing with HeLaT4+ cells. The reversion of the inhibitory effect was achieved by pre-incubating the MAb with peptide-containing vesicles for 30 min before adding it to the CHO-Env cells.

### Electron Microscopy

For preparing cryo-TEM samples, peptide-containing liposomes and MAb were mixed under continuous stirring. After incubation, drop samples were collected from the mixtures and applied to holey carbon films, quickly blotted and frozen in liquid ethane at −180°C, so that the vesicles were preserved in vitreous ice. The frozen grids were kept under liquid nitrogen until used. Images were acquired with a defocus range of 1.5–2.0 µm on an 4 K Å∼4 K Eagle CCD camera (Gatan Inc.) mounted on a FEI Tecnai G2 FEG200 electron microscope at 200 kV with a Gatan side-entry and a GATAN anticontaminator.

### Rabbit Immunization and Response Analysis

Immunizations with liposomal formulations were carried out at the Antibody Production Service from the CID-CSIC (Barcelona, Spain), according to the method described by Dressman et al. [81]. MPERp:FPp mixtures preincubated in DMSO were added at a final peptide-to-lipid ratio of 1∶50 (mol:mol) to a stirring solution of freeze-thaw POPC:Chol:PA (2.0∶1.5∶0.2 molar ratio) vesicles dispersed in PBS. PA was included in the lipid composition for increasing vesicle stability in serum [81]. Control experiments revealed that this additional bilayer component did not affect recognition of membrane-inserted epitopes by the MAb-s appreciably (not shown). After incubation for 30 minutes, the samples were lyophilized. New Zealand White rabbits were inoculated intradermally at multiple sites on day 0 with 1 ml of sample reconstituted in pure water, which contained 0.5 mg peptide supplemented with 1.25 mg of muramyl dipeptide (MDP) (Sigma-Aldrich, St-Louis, MO). For subsequent boosting injections, 1 ml of the reconstituted liposome formulation containing 0.3 mg peptide was used on day 15 (0.3 mg peptide), while 0.2 mg of liposomal peptide were injected on days 30, 45 and 60. The 2F5 epitope-specific antibodies were recovered from sera through affinity purification. To that end, 2F5ep-Cys (Table 1) was immobilized onto a beaded agarose support using a Sulfolink Immobilization Kit for Peptides (Thermo Scientific, Rockford, IL) and following the manufacturer’s instructions. The remaining nonspecific binding sites in columns were blocked adding L-Cysteine·HCl at 50 mM. Every analyzed serum was loaded on the columns after diluting and filtering it to remove the particulate material. They were let flow through the columns 5 times allowing the binding of all the antibodies present in the serum that recognize specifically the immobilized peptide. After washing the columns with at least 10 bed volumes of 500 mM NaCl containing buffer to dispose of nonspecifically bound antibodies and serum proteins, the specific antibodies (Bound) were eluted using 100 mM Glycine buffer at pH 2.5. The fraction that is not recovered using acidic pH was eluted using freshly made 100 mM Triethylamine buffer at pH 11.5.

One set of neutralization assays was performed using an AA-2 cell and infectious virus (HIV-1 IIIB) as described previously [5]. Briefly, 2-fold dilution series of antibodies were preincubated with virus at 10^2^–10^3^ 50% tissue culture infective dose (TCID_50_)/ml for 1 h at 37°C. CD4-positive human AA-2 cells (obtained through the ARRRP-NIH, contributed by Dr. M. S. Hershfield) were then added at a cell count of 4×10^5^ cells/ml and further incubated for 5 days. The read-out was performed according to the method of Reed and Muench, and the presence of at least one syncytium was scored positive.

Another set of neutralization assays was carried out using TZM-bl cells and HIV-1 pseudoviruses as previously described [35], [38]. Pseudoviruses were produced by transfection of human kidney HEK293T cells with the full-length env clone pHXB2-env (AIDS Research and Reference Reagent Program, Division of AIDS, NIAID, NIH, contributed by K. Page and D. Littman) using calcium phosphate, together with vectors pWXLP-GFP and pCMV8.91, encoding respectively a green fluorescent protein and an env-deficient HIV-1 genome (kindly provided by Dr. Patricia Villace, CSIC, Madrid). After 24 h, the medium was replaced with Optimem-Glutamax II (Invitrogen Ltd, Paisley, UK) without serum. Two days after transfection, the pseudovirus particles were harvested, passed through 0.45 µm pore sterile filters (Millex® HV, Millipore NV, Brussels, Belgium) and finally concentrated by ultracentrifugation in a sucrose gradient. Neutralization was determined using TZM-bl target cells. Samples were set up in duplicate in 96-well plates, and incubated for 1 h at 37°C with a 10–15% tissue culture infectious dose of pseudovirus. After IgG-pseudovirus co-incubation, 10,000 target cells were added in the presence of 15 µg/ml DEAE-dextran (Sigma-Aldrich, St-Louis, MO). Neutralization levels after 72 hours were inferred from the reduction in the number of GFP-positive cells as determined by flow cytometry using a BD FACSCalibur Flow Cytometer (Becton Dickinson Immunocytometry Systems, Mountain View, CA).

## Supporting Information

Figure S1 **MPER/FP complex recognition by Dot-blot.** Left: decreasing amounts of peptides (from top to bottom: 34, 23, 15, 10, 6.75, 4.5, 3, 2, 1.3 pmol) were spotted onto Hybond C nitrocellulose, and allowed to dry. The nitrocellulose was then blocked with 1% fat-free milk in PBS (Blocking Buffer) for 1 h and incubated for 1 more hour with MAb2F5 (0.1 µg/ml) in Blocking Buffer at room temperature. The membranes were washed 3 times, 10 min each with PBS, and soaked in Blocking Buffer with horseradish peroxidase-conjugated anti mouse human antibody (GE Healthcare) at a 1∶2000 dilution for 1 h at room temperature. After washing with PBS 3×10 min, the MAb2F5 was detected by chemiluminescence. Right: peptides were co-solubilized with POPC:Chol (2∶1) lipid mixtures (1∶100 peptide-to-lipid mole ratio) in 2∶1:0.8 MeOH:CHCl_3_:H_2_O, and subjected to the same procedure described above. LipidCTL designates the only lipid control.
(TIFF)

Figure S2 **Membrane-bound MAb2F5 particles as detected by cryo-TEM.** Micrographs correspond to LUV pre-incubated with MPERp:FPp mixture and antibody as indicated in the caption for Figure 6 (left panel). Arrows point to rods protruding from the membrane surface. The scale bar represents 100 nm.
(TIFF)

Figure S3 **MAb2F5-induced effects on MPERp/FPp-containing lipid vesicle morphology.** Micrographs correspond to LUV pre-incubated with MPERp:FPp mixture and antibody as indicated in the caption for Figure 6 (right panel). A) The fields show vesicle aggregation induced by the antibody. Tubular structures and apparent loss of the bilayer integrity (open vesicles) can be observed at some points. The scale bar represents 100 nm. B–F) Antibody particles (indicated by arrows and asterisks) concentrated at the surface of vesicles that displayed morphologies consistent with loss of bilayer integrity (red dotted lines) and membrane evagination (blue bilayers).
(PDF)

Figure S4 **Immunogenicity of membrane-bound peptides.** A) Sera obtained from two rabbits (59 and 60) immunized with membrane-bound MPERp/FPp were titrated in ELISA using the MPERp/FPp mixture (1.4 µM of each peptide). Black symbols represent the respective pre-immune sera. B) Cross-reactivity of the sera to 1.4 μΜ MPERp (black), rec-gp41 (blue), 2F5ep (green), preTM (red) and C34 (brown) immobilized in ELISA plates.
(TIF)

Figure S5 **Evidence for specific structures adopted by membrane-bound MPERp/FPp complexes.** A) Left: Circular dichroism (CD) spectra of the MPERp:FPp mixture in the presence of POPC:Chol vesicles. The panel displays the comparison of the experimental spectrum (solid line) and the spectrum calculated for the addition of non-interacting peptide signals (dotted line). The significant differences between these spectra are consistent with a conformational rearrangement of the MPERp:FPp mixtures upon contact with vesicles. Right: comparable experimental spectra were measured for the complex (solid line) in the presence of 5% of the structure-promoting HFIP. Previous structural characterization of HybK3, a hybrid peptide combining FP and 2F5 epitope sequences, indicated that conformers containing high proportion of type I β-turns could give rise to this kind of CD spectra [22]. To illustrate this point the CD spectrum of HybK3 is displayed in the same panel (dashed line). B) Left: the experimental and calculated spectra for MPERp:FPp mixtures coincided in dodecylphosphocholine (DPC) micelles. These spectra were compatible with the adoption of main α-helical conformations by each peptide. Right: comparable spectra could also be recovered in the low-polarity medium provided by 25% HFIP. C) Cross-linking assays. Cross-linking was much more effective if the peptides were stored with vesicles (left panel), than if they were solubilized by DPC (right panel).
(PDF)

Figure S6 **Recovery from rabbit serum of antibodies targeting MPERp/FPp complex on vesicle surfaces.** A) Peptide recognition by rabbit IgG purified with MPERp/FPp-Cys17 adsorbed onto vesicle surfaces. Recovered antibodies were titrated by ELISA against the following peptides: FPp (blue); C34 (orange); MPERp (red); CpreTM (green); TMDp (black). Reactivity to 2F5ep is denoted by empty red symbols and dotted line. B) Neutralization assays with rabbit IgG purified with 2F5ep-Cys or MPERp/FPp-Cys17 on vesicles (red and blue symbols, respectively). In these assays, HXB2-env pseudoviruses were pre-incubated with antibodies, and infection of TZM-bl target cells subsequently monitored by flow cytometry as previously described [35], [38]. Plotted infection percentage values are means of two experimental determinations.
(TIFF)
