# Supplementary figures and images for: Recognition of Membrane-Bound Fusion-Peptide/MPER Complexes by the HIV-1 Neutralizing 2F5 Antibody: Implications for Anti-2F5 Immunogenicity
Source: PLoS One. 2012 Dec 21;7(12):e52740. doi: 10.1371/journal.pone.0052740 (PMC3528738; doi:10.1371/journal.pone.0052740)

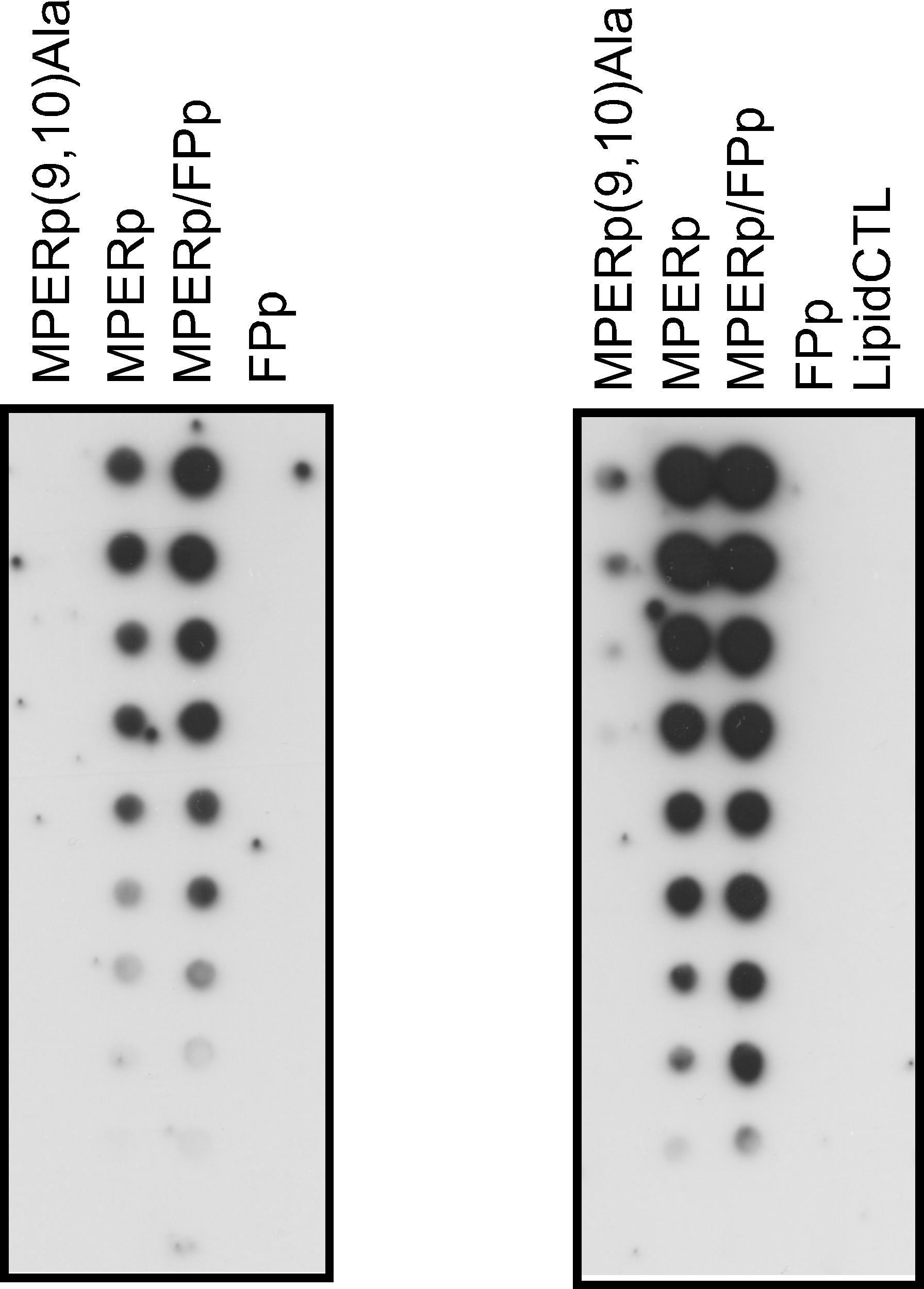

Supplement: Figure S1 — MPER/FP complex recognition by Dot-blot. Left: decreasing amounts of peptides (from top to bottom: 34, 23, 15, 10, 6.75, 4.5, 3, 2, 1.3 pmol) were spotted onto Hybond C nitrocellulose, and allowed to dry. The nitrocellulose was then blocked with 1% fat-free milk in PBS (Blocking Buffer) for 1 h and incubated for 1 more hour with MAb2F5 (0.1 µg/ml) in Blocking Buffer at room temperature. The membranes were washed 3 times, 10 min each with PBS, and soaked in Blocking Buffer with horseradish peroxidase-conjugated anti mouse human antibody (GE Healthcare) at a 1∶2000 dilution for 1 h at room temperature. After washing with PBS 3×10 min, the MAb2F5 was detected by chemiluminescence. Right: peptides were co-solubilized with POPC:Chol (2∶1) lipid mixtures (1∶100 peptide-to-lipid mole ratio) in 2∶1:0.8 MeOH:CHCl3:H2O, and subjected to the same procedure described above. LipidCTL designates the only lipid control. (TIFF) [file pone.0052740.s001.tif]

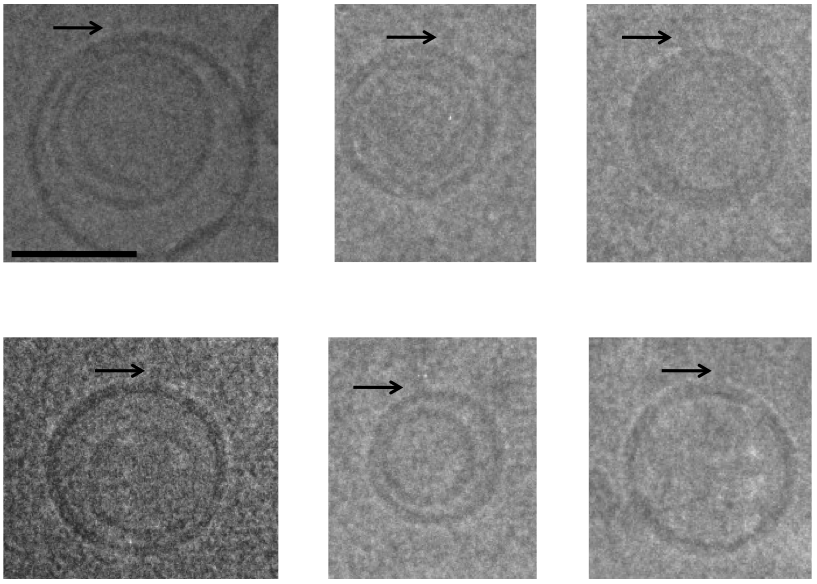

Supplement: Figure S2 — Membrane-bound MAb2F5 particles as detected by cryo-TEM. Micrographs correspond to LUV pre-incubated with MPERp:FPp mixture and antibody as indicated in the caption for Figure 6 (left panel). Arrows point to rods protruding from the membrane surface. The scale bar represents 100 nm. (TIFF) [file pone.0052740.s002.tif]

A

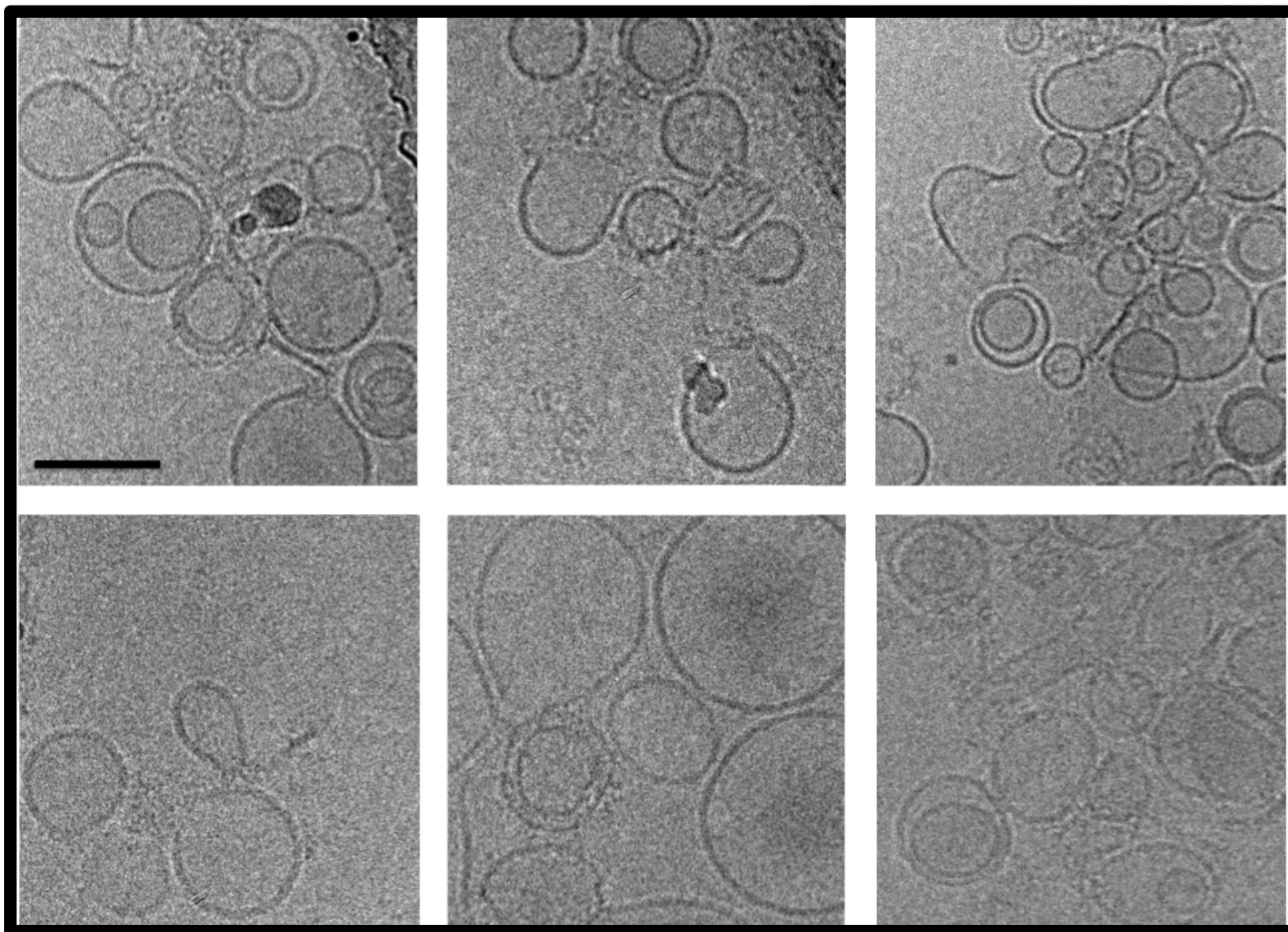

B

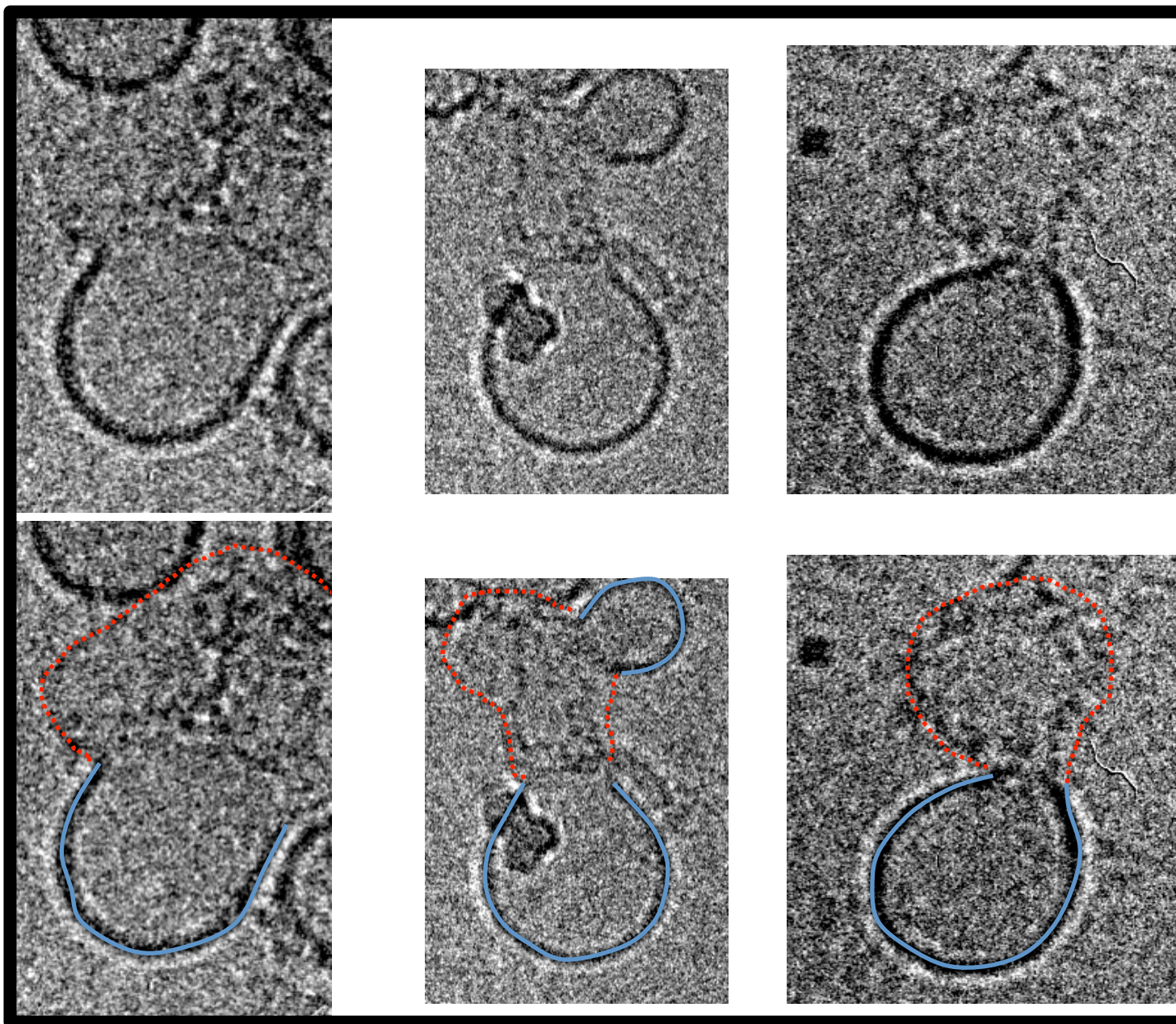

C

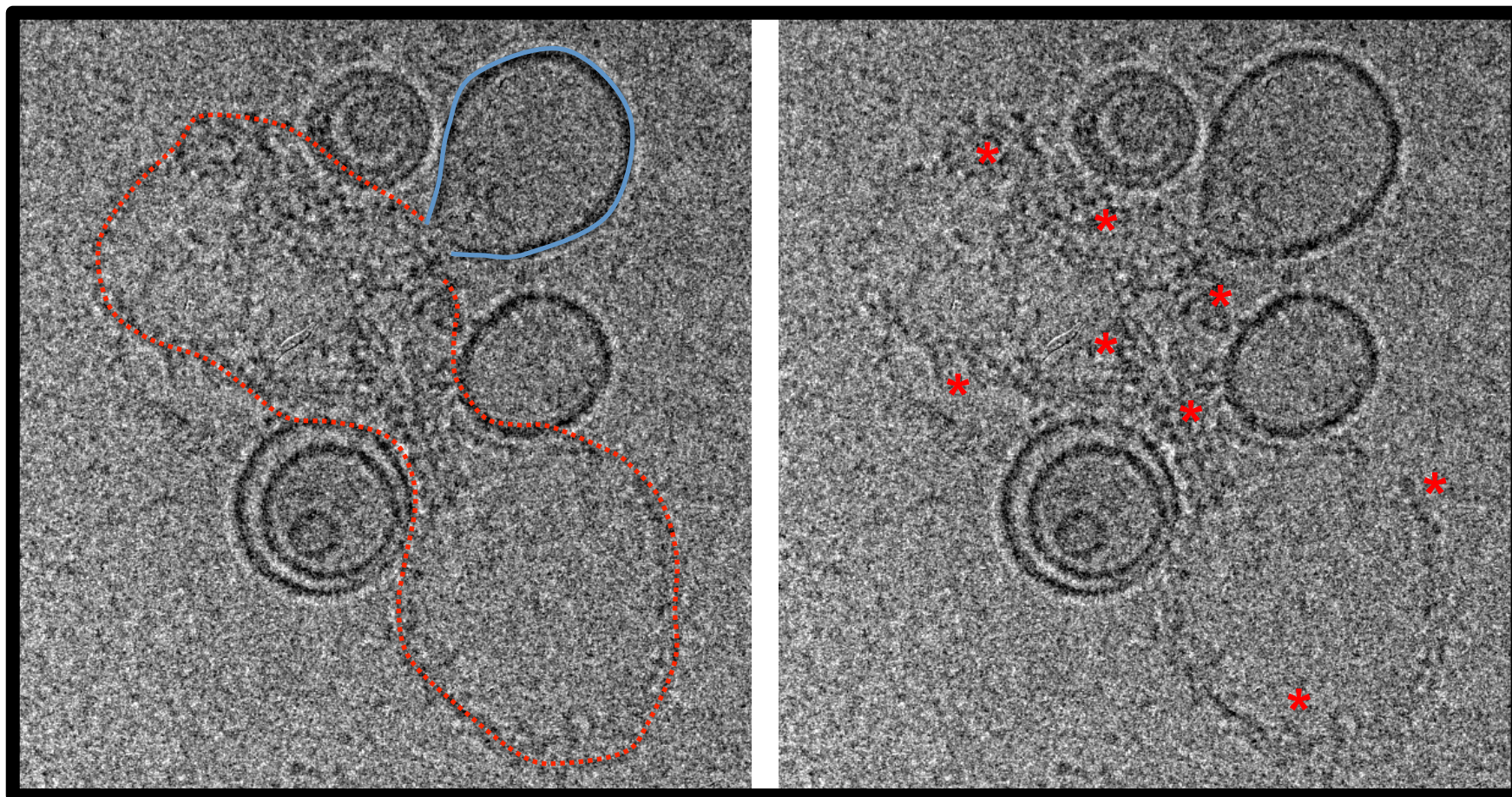

D

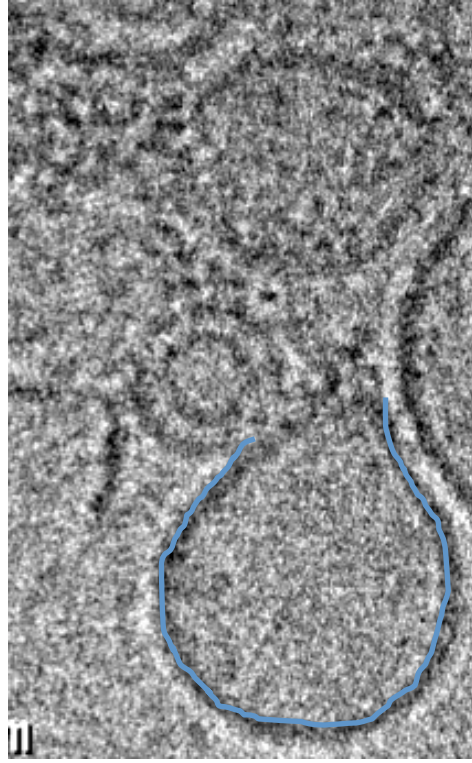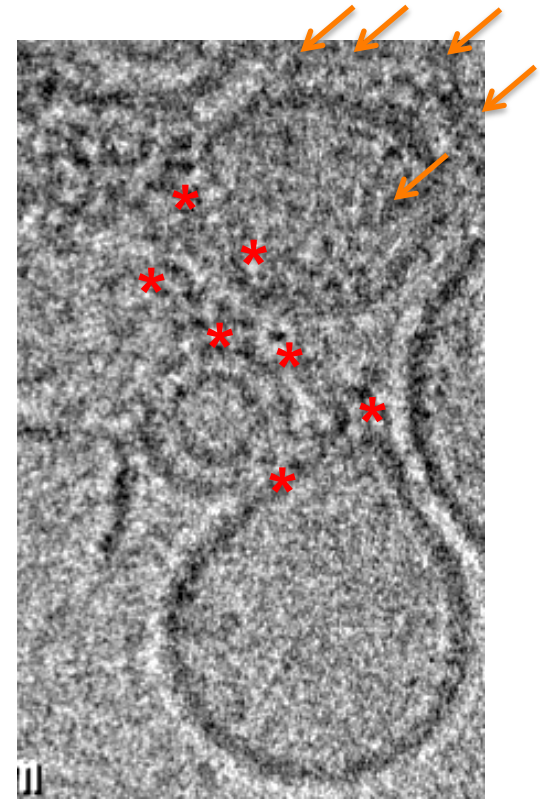

E

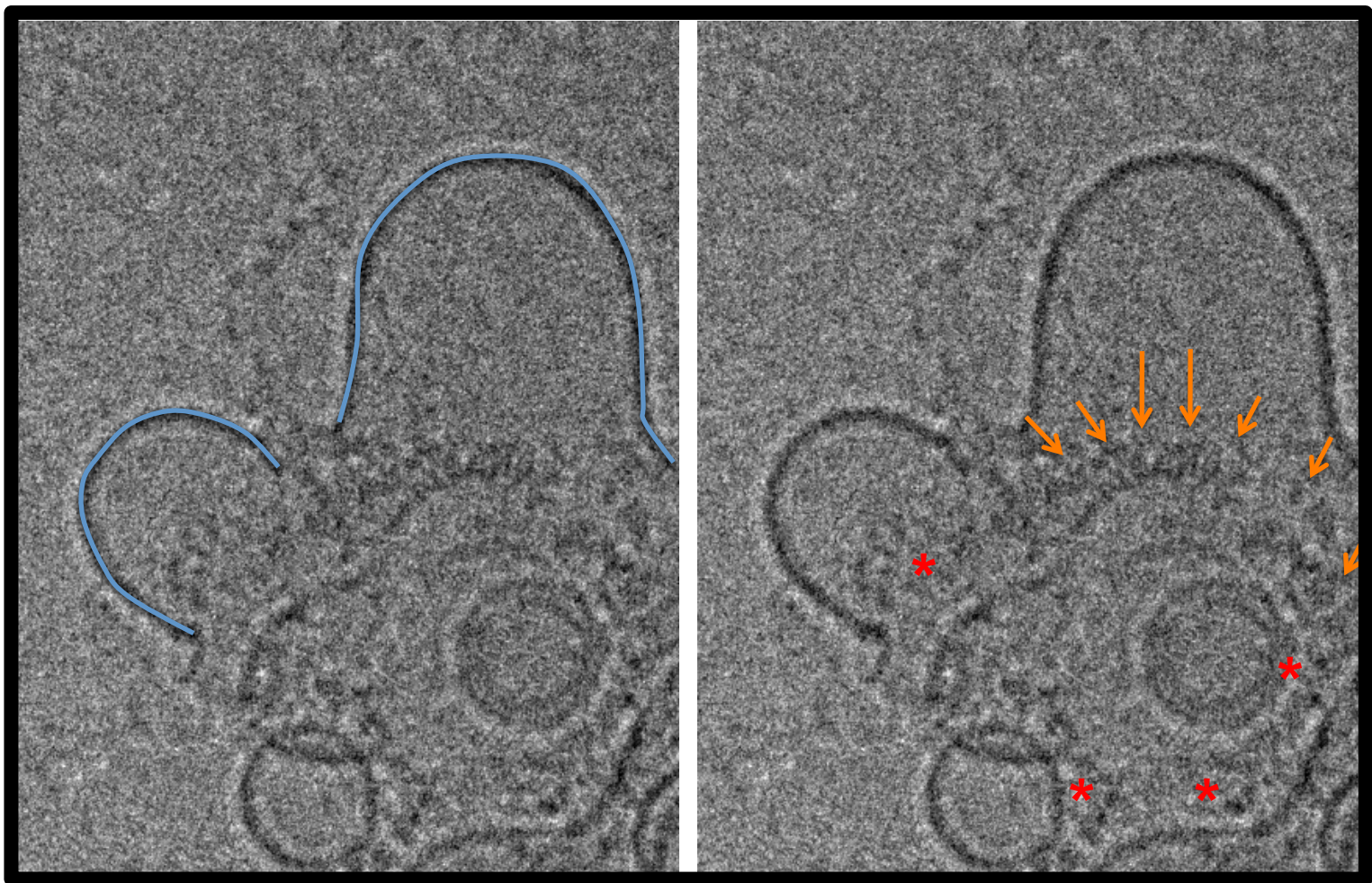

F

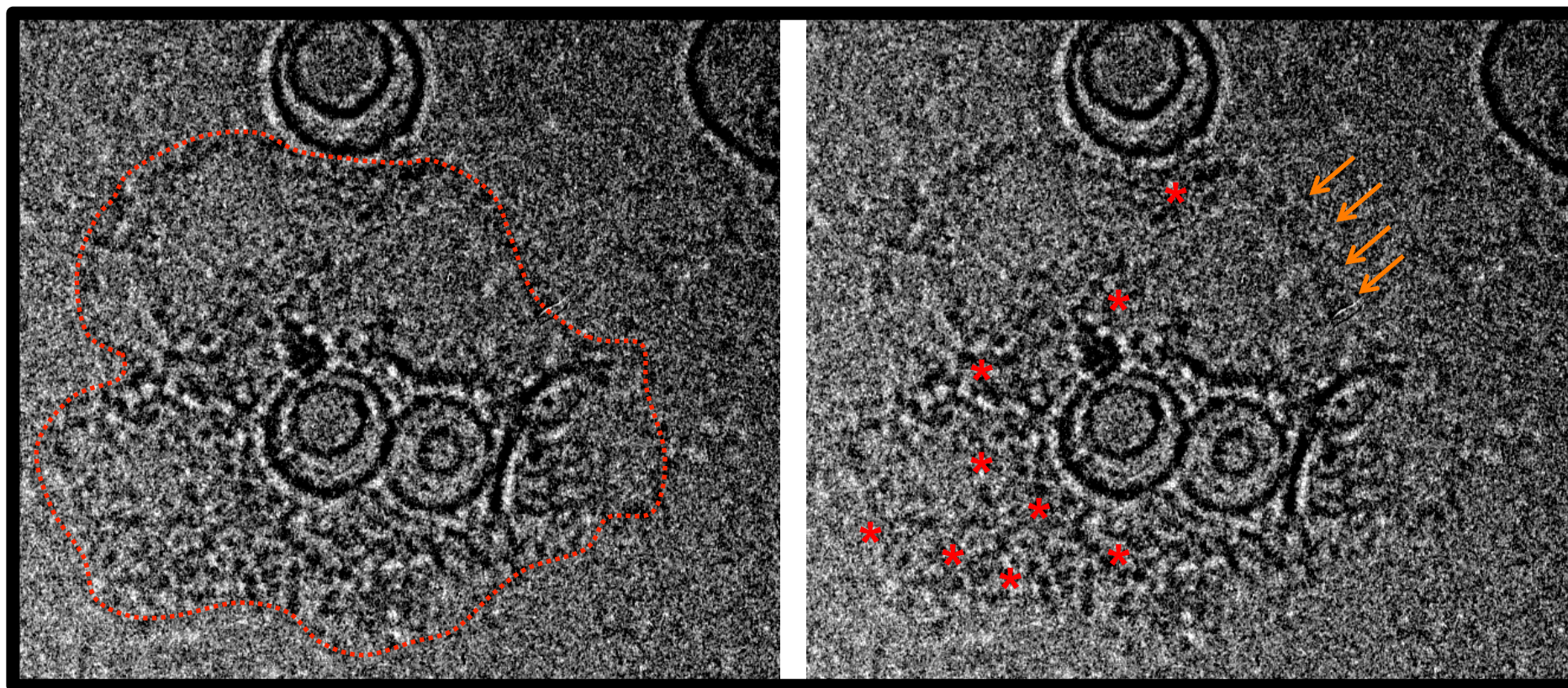

Supplement: Figure S3 — MAb2F5-induced effects on MPERp/FPp-containing lipid vesicle morphology. Micrographs correspond to LUV pre-incubated with MPERp:FPp mixture and antibody as indicated in the caption for Figure 6 (right panel). A) The fields show vesicle aggregation induced by the antibody. Tubular structures and apparent loss of the bilayer integrity (open vesicles) can be observed at some points. The scale bar represents 100 nm. B–F) Antibody particles (indicated by arrows and asterisks) concentrated at the surface of vesicles that displayed morphologies consistent with loss of bilayer integrity (red dotted lines) and membrane evagination (blue bilayers). (PDF) [file pone.0052740.s003.pdf]

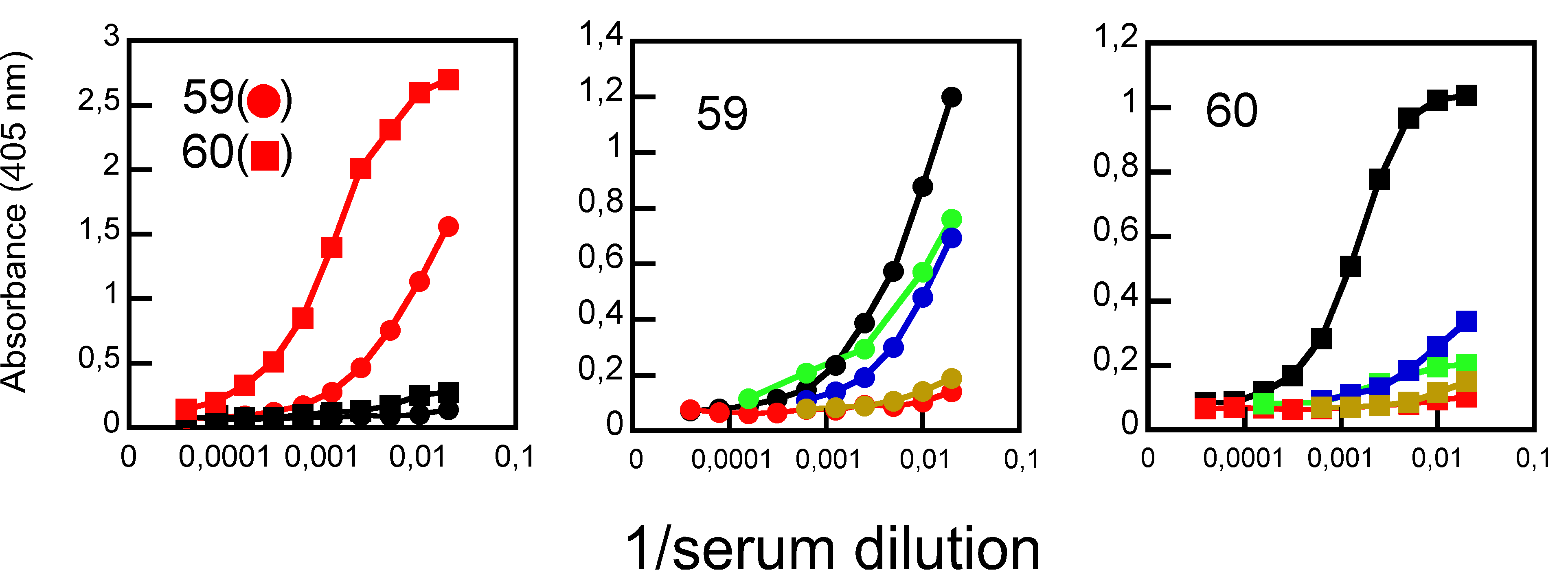

Supplement: Figure S4 — Immunogenicity of membrane-bound peptides. A) Sera obtained from two rabbits (59 and 60) immunized with membrane-bound MPERp/FPp were titrated in ELISA using the MPERp/FPp mixture (1.4 µM of each peptide). Black symbols represent the respective pre-immune sera. B) Cross-reactivity of the sera to 1.4 μΜ MPERp (black), rec-gp41 (blue), 2F5ep (green), preTM (red) and C34 (brown) immobilized in ELISA plates. (TIF) [file pone.0052740.s004.tif]

A

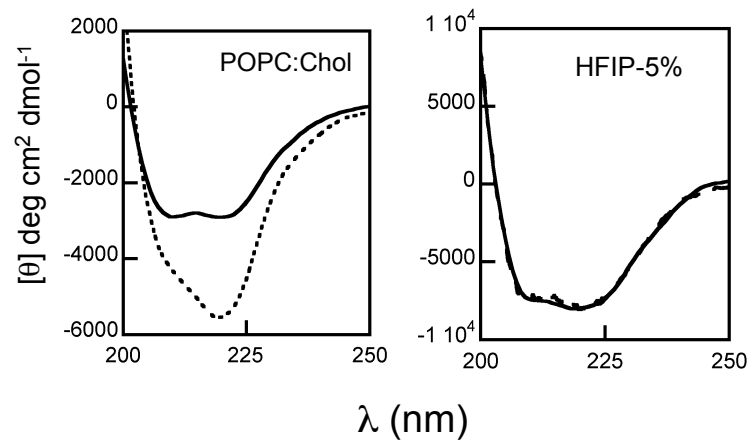

B

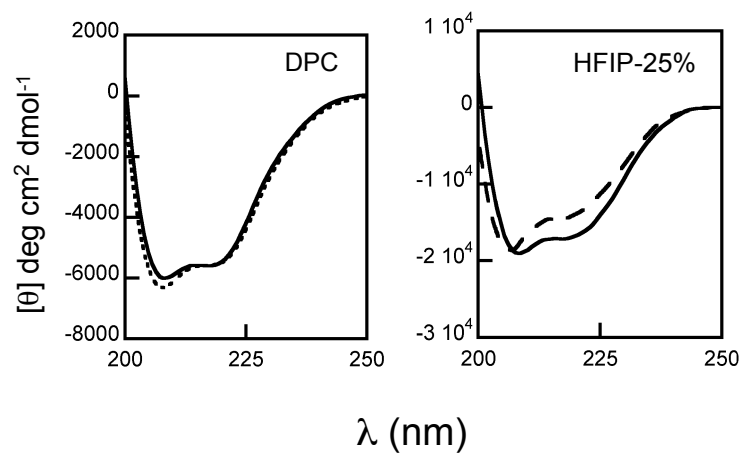

C

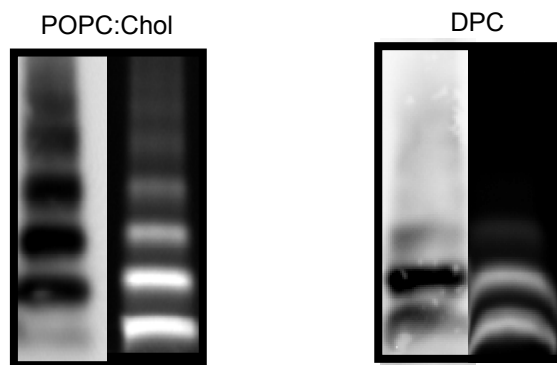

Supplement: Figure S5 — Evidence for specific structures adopted by membrane-bound MPERp/FPp complexes. A) Left: Circular dichroism (CD) spectra of the MPERp:FPp mixture in the presence of POPC:Chol vesicles. The panel displays the comparison of the experimental spectrum (solid line) and the spectrum calculated for the addition of non-interacting peptide signals (dotted line). The significant differences between these spectra are consistent with a conformational rearrangement of the MPERp:FPp mixtures upon contact with vesicles. Right: comparable experimental spectra were measured for the complex (solid line) in the presence of 5% of the structure-promoting HFIP. Previous structural characterization of HybK3, a hybrid peptide combining FP and 2F5 epitope sequences, indicated that conformers containing high proportion of type I β-turns could give rise to this kind of CD spectra [22]. To illustrate this point the CD spectrum of HybK3 is displayed in the same panel (dashed line). B) Left: the experimental and calculated spectra for MPERp:FPp mixtures coincided in dodecylphosphocholine (DPC) micelles. These spectra were compatible with the adoption of main α-helical conformations by each peptide. Right: comparable spectra could also be recovered in the low-polarity medium provided by 25% HFIP. C) Cross-linking assays. Cross-linking was much more effective if the peptides were stored with vesicles (left panel), than if they were solubilized by DPC (right panel). (PDF) [file pone.0052740.s005.pdf]

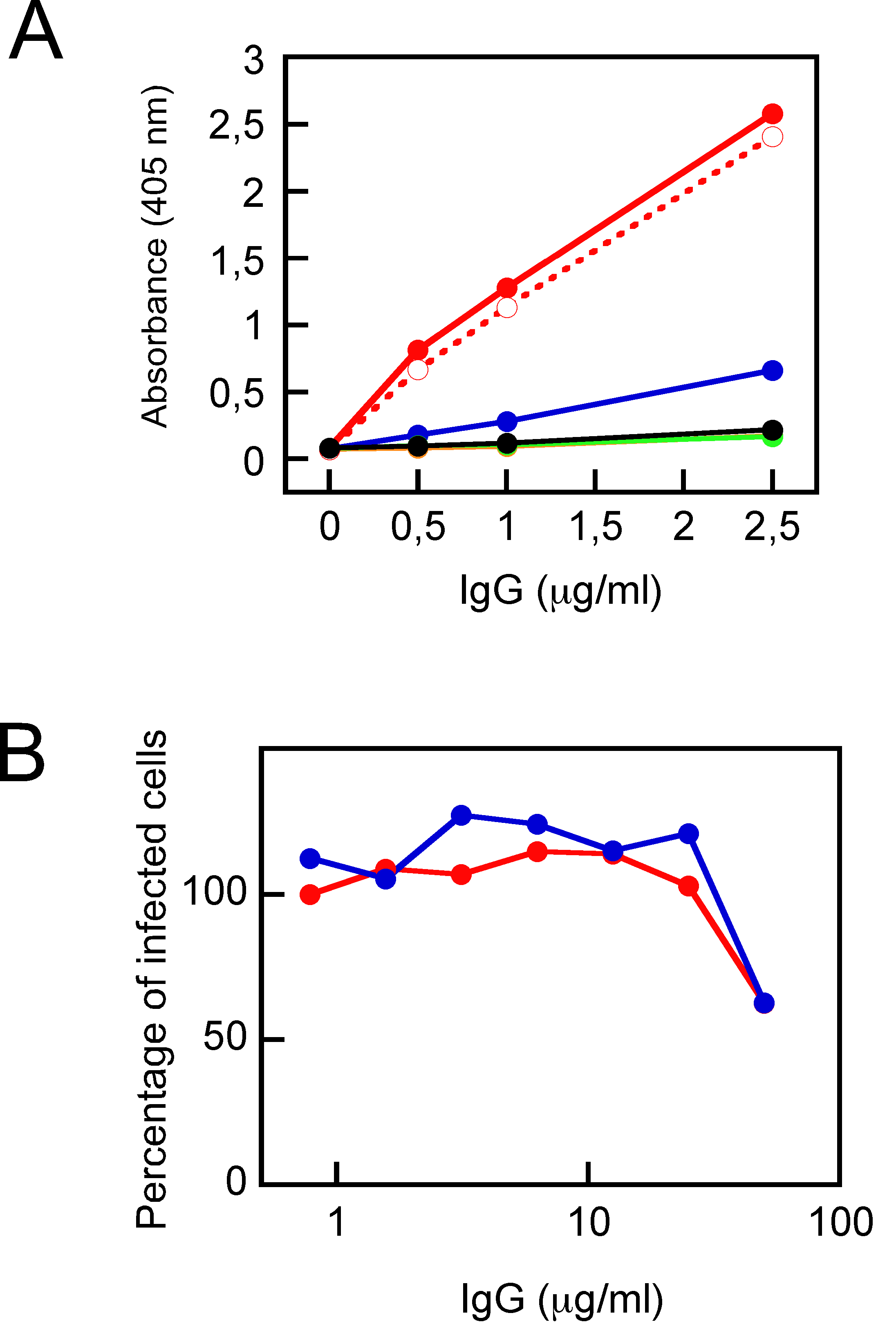

Supplement: Figure S6 — Recovery from rabbit serum of antibodies targeting MPERp/FPp complex on vesicle surfaces. A) Peptide recognition by rabbit IgG purified with MPERp/FPp-Cys17 adsorbed onto vesicle surfaces. Recovered antibodies were titrated by ELISA against the following peptides: FPp (blue); C34 (orange); MPERp (red); CpreTM (green); TMDp (black). Reactivity to 2F5ep is denoted by empty red symbols and dotted line. B) Neutralization assays with rabbit IgG purified with 2F5ep-Cys or MPERp/FPp-Cys17 on vesicles (red and blue symbols, respectively). In these assays, HXB2-env pseudoviruses were pre-incubated with antibodies, and infection of TZM-bl target cells subsequently monitored by flow cytometry as previously described [35], [38]. Plotted infection percentage values are means of two experimental determinations. (TIFF) [file pone.0052740.s006.tiff]
